# Sedentary Behavior and Health Outcomes: An Overview of Systematic Reviews

**DOI:** 10.1371/journal.pone.0105620

**Published:** 2014-08-21

**Authors:** Leandro Fornias Machado de Rezende, Maurício Rodrigues Lopes, Juan Pablo Rey-López, Victor Keihan Rodrigues Matsudo, Olinda do Carmo Luiz

**Affiliations:** 1 Departamento de Medicina Preventiva, Faculdade de Medicina da Universidade de São Paulo, São Paulo, Brasil; 2 Centro de Estudos do Laboratório de Aptidão Fĩsica de São Caetano do Sul, São Caetano do Sul, Brasil; Universidad Europea de Madrid, Spain

## Abstract

**Objective:**

1) To synthesize the current observational evidence for the association between sedentary behavior and health outcomes using information from systematic reviews. 2) To assess the methodological quality of the systematic reviews found.

**Methodology/Principal Findings:**

Medline; *Excerpta Medica* (Embase); PsycINFO; and Web of Science were searched for reviews published up to September 2013. Additional publications were provided by Sedentary Behaviour Research Network members. The methodological quality of the systematic reviews was evaluated using recommended standard criteria from AMSTAR. For each review, improper use of causal language in the description of their main results/conclusion was evaluated. Altogether, 1,044 review titles were identified, 144 were read in their entirety, and 27 were included. Based on the systematic reviews with the best methodological quality, we found in children and adolescents, strong evidence of a relationship between time spent in sedentary behavior and obesity. Moreover, moderate evidence was observed for blood pressure and total cholesterol, self-esteem, social behavior problems, physical fitness and academic achievement. In adults, we found strong evidence of a relationship between sedentary behavior and all-cause mortality, fatal and non-fatal cardiovascular disease, type 2 diabetes and metabolic syndrome. In addition, there is moderate evidence for incidence rates of ovarian, colon and endometrial cancers.

**Conclusions:**

This overview based on the best available systematics reviews, shows that sedentary behavior may be an important determinant of health, independently of physical activity. However, the relationship is complex because it depends on the type of sedentary behavior and the age group studied. The relationship between sedentary behavior and many health outcomes remains uncertain; thus, further studies are warranted.

## Introduction

Physical inactivity, or lack of moderate to vigorous physical activity, [1] is strongly related to the main non-communicable diseases such as coronary heart disease, [2] type 2 diabetes [3] and certain types of cancer. [4] In addition, many studies have demonstrated that physical inactivity is an important determinant of all-cause mortality. [5], [6].

However, recently a new paradigm in the physical activity field has emerged. [7] Many epidemiological studies have consistently shown that spending excessive time engaged in sedentary behaviors may have a negative impact on several health outcomes, independently of moderate to vigorous physical activity. [8], [9] Sedentary behavior is defined as time spent engaged in sitting or lying down activities that require an energy expenditure of 1.0 to 1.5 basal metabolic rates. [10] Sedentary activities are described in different domains, such as work, leisure/entertainment and commuting.[11]–[13] In addition, these activities have been categorized as nondiscretionary or discretionary. Behaviors such as sitting at work, school or while commuting via car or bus are nondiscretionary, whereas watching television, reading, using a computer, and playing video games are discretionary. [14].

During the last decade, a growing number of systematic reviews have been published.[15]–[41] However, most of them have focused on one particular sedentary behavior (i.e. television viewing), age group or health outcome and have drawn divergent conclusions. Therefore, an overview of systematic reviews is needed to cover all types of sedentary behavior, health outcomes and age groups, taking into account the methodological quality of the systematic reviews. This overview method has been used in medical and behavioral studies. [42], [43].

Thus, the aim of this overview was to synthesize the current evidence of the relationship between sedentary behavior and health outcomes during the time periods reported in the systematic reviews. Moreover, for each systematic review a methodological quality assessment was performed.

## Methods

### Criteria for considering reviews for inclusion

To be included in our overview, reviews had to describe the search methods used and the inclusion criteria of the original articles.

### Article selection

A comprehensive search was performed up until September 2, 2013 using Medline; *Excerpta Medica* (Embase); PsycINFO; and Web of Science. Keywords related to exposure (sedentary behavior, sedentary lifestyles, sedentary time, sitting time, television viewing, driving, screen-based, video game, computer, and screen time) and method (“systematic review” and “meta-analysis”) were included in the search. Detailed information on the combinations of search terms used in our search strategy is shown in File S1. The systematic reviews retrieved were imported into the EndNote Web reference management software (Thomson Reuters, Carlsbad, CA, USA). All eligible articles were evaluated by two independent reviewers, who examined all of the empirical evidence and discussed the discrepancies. Disagreements between the two reviewers were settled by a third reviewer. Reference lists in the selected systematic reviews and approximately 400 individuals affiliated with the Sedentary Behaviour Research Network (professors, researchers, and students), were contacted in an attempt to identify more articles for inclusion in our overview.

### Inclusion and exclusion criteria

To be included in the overview, articles had to be systematic reviews, with or without a meta-analysis that examined the relationship between sedentary behavior and health outcomes among observational studies. We excluded the following types of reviews: reviews in which sedentary behavior was inappropriately defined (as if it were synonymous with physical inactivity, i.e. failing to meet the minimum physical activity recommendations); and narrative reviews of the literature. Because of the large number of review articles initially selected, we excluded those examining other aspects of sedentary behavior i.e. interventions to reduce sedentary behavior; determinants/correlates of sedentary behavior; the tracking of sedentary behavior; and different methods for measuring sedentary behavior.

### Data extraction

All eligible systematic reviews included were examined and extracted independently by two reviewers (LFMR and MRL). The data extracted included information on author(s), year, age group, type of sedentary behavior, outcome measure, whether a meta-analysis was conducted, and quality assessment of the original studies (File S2), eligibility criteria and evaluation of physical activity as a covariate (File S3).

### Quality Assessment of Systematic Reviews

All included reviews were evaluated by two independent reviewers (LFMR and JPRL) using the Assessing the Methodological Quality of Systematic Reviews (AMSTAR) tool (File S4). [44], [45] AMSTAR contains 11-items to appraise the methodological aspects of the systematic reviews. These items are described in the File S4. The score for each item was determined as: yes = 1 point and no/N/A = 0. Therefore, the total score could range from 0 to 11.

### Level of Scientific Evidence

The level of evidence for each health outcome was classified as *strong, moderate, insufficient or no evidence* (File S5). To determine the level of evidence for each health outcome, we first selected the best systematic reviews according to the AMSTAR score. Secondly, conclusions of these systematic reviews were maintained if considered the methodological quality of the included studies. Finally, reviews must took into account several covariates (especially physical activity).

If the best systematic reviews did not take into account any of these additional items, we decreased the level of the evidence reported to the next lower level. For example, if a review had a *strong level of evidence* and it did not include the above criteria, we then classified it as a *moderate level of evidence*.

### Use of causative language

For each review, improper use of causal language in the description of their main results/conclusion was evaluated (File S6). A review was rated as causal if causal language was used (i.e., “Low sedentary behavior is *protective* of obesity”). Reviews were rated as qualified causal if words such as “*may*” or “*suggest*” were included to describe their main results. Finally, reviews were considered acceptable if the inference was based on *associations* or *relationships*. This methodology has been used by Brown et al. [46].

### Ethics

No ethical approval was required.

## Results

**Figure 1:**
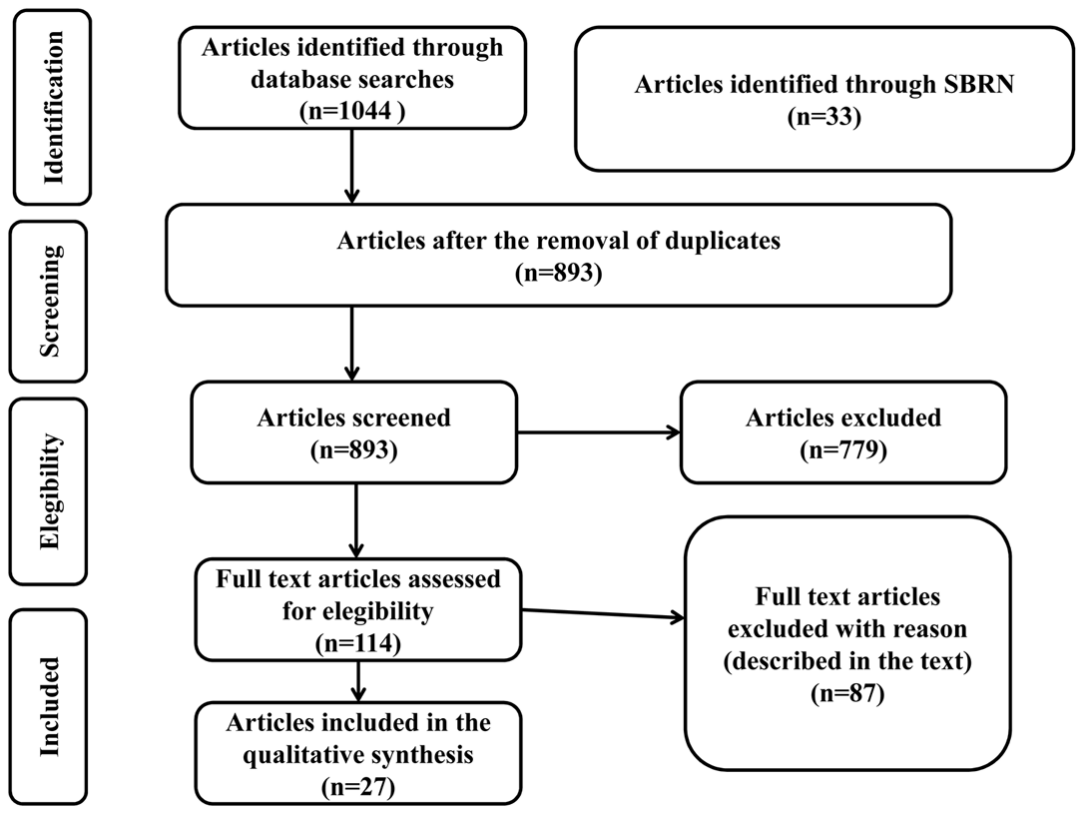
Preferred Reporting Items for Systematic Reviews flow diagram of the studies included in our overview.

A total of 1044 potentially relevant articles were initially retrieved from the databases searched. Of those 1044 articles, 424 were retrieved from Medline, 248 from Embase, 333 from Web of Science, and 39 from PsycINFO. (Figure 1) Another 33 articles were included, that were selected from among the titles suggested by our Sedentary Behaviour Research Network contacts. After the exclusion of duplicate entries, 893 articles remained. After screening the titles and abstracts, we selected 114 articles to be read in their entirety. Of those, only 27 met the criteria for inclusion in our overview.

We found no review articles examining sedentary behavior in middle-aged adults or in the elderly (individuals over 65 years of age). As shown in File S2, we included 13 reviews investigating sedentary behavior in children and adolescents (0–18 years of age).[15]–[27]. In addition, we included 8 articles investigating sedentary behavior in adults over 18 years of age,[21], [28]–[34] and 7 articles investigating sedentary behavior in a variety of age groups.[35]–[41] One article addressed the topic in adults and in children. [21] Consequently, that study was included in both categories and was, therefore, counted twice. One review [47] about sedentary behavior and schizophrenia and bipolar disorder was identified in our search strategy, but it was excluded in our overview because no sedentary behavior studies were found.

File S2 shows the reviews included in the overview (n = 27). Reviews were published between 2004 [19] and 2013 [16], [17], [39]. The quality of the original studies was assessed in 16 (60%) reviews,[15], [16], [18], [20], [21], [26], [27], [28], [31], [32], [34], [36], [38]–[41], and 6 (22%) performed a meta-analysis. [19], [26], [28], [29], [34], [37].

Among the 27 systematic reviews, 17 did not restrict the type of sedentary behavior in the research question,[15], [17], [18], [21]–[23], [25]–[28], [30]–[35], [39] 8 used only screen-based sedentary behavior, [16], [19], [20], [24], [29], [37], [38], [41], and 3 used sitting-time (at work or at leisure). [36], [37], [40].

In general, the systematic reviews’ eligibility criteria were based on age, language, date published, and study design (File S3). Physical activity was evaluated as a covariate in 11 (41%) systematic reviews. [15], [16], [28], [29], [30], [31], [33], [34], [37], [40], [41] Of those, the proportion of original studies that included physical activity as a covariate ranged between 15%–100% (mean 63%). The information on whether physical activity was assessed objectively or by questionnaire was only available in Edwardson et al. (7 studies with a questionnaire and 1 with an accelerometer) [28], and Ford and Caspersen (11 studies with a questionnaire and 0 with an accelerometer) [37] systematic reviews (File S3).

### Methodological Quality

Based on AMSTAR, we assessed the methodological quality of the 27 reviews included in the overview (see File S4). For children and adolescents, 6 reviews (46%) scored ≥6 points. [15], [16], [18], [20], [26], [27]. In adults, 5 reviews (62%) scored ≥6 points, [28], [29], [31], [32], [34] whereas the 3 reviews with unspecified ages (43%) had a total score ≥6 points. [38], [39], [41] Additionally, the quality assessment of the included articles was conducted in 17 (63%) reviews,[15], [16], [18], [20], [21], [25], [27], [28], [31], [32], [34]–[36], [38]–[41] and 16 (60%) used the quality appropriately to formulate conclusions.[15], [16], [18], [20], [21], [25], [27], [28], [31], [32], [34]–[36], [38], [40], [41].

### Outcomes

The level of scientific evidence synthesized for each outcome according to age group and sedentary behavior type is shown in the File S5.

#### Mortality

Seven systematic reviews investigated the association between sedentary behavior and mortality in adults.[29]–[31], [33], [34], [37], [40] Consistent findings of prospective studies and studies with high levels of methodological quality suggest that sedentary behavior is associated with all-cause and cardiovascular mortality, regardless of the level of physical activity and body mass index (BMI). [29], [31], [33], [34], [37], [40].

In Ford et al., [37] for each 2-hours of additional sitting time there was a 5% increase in cardiovascular mortality (HR 1.05; 95% CI 1.01–1.09). Grøntved and Hu [29] found that watching television for more than 2 hours per day was associated with a 13% increase in all-cause mortality (RR, 1.13; 95% CI, 1.07–1.18).

According to Wilmot et al., [34] for adults that spend most of their time engaged in sedentary behaviors (screen-time and sitting time), compared to those who spend very little time engaged in such behaviors, the relative risk for all-cause mortality and cardiovascular mortality is 1.49 (95% CI, 1.14–2.03) and 1.90 (95% CI, 1.36–2.66), respectively. However, most of the studies evaluated by Wilmot et al. [34] were cross-sectional studies that did not employ standardized measures of the time spent in sedentary behavior, which would have allowed the summary measure to have been calculated in the meta-analysis.

Although some systematic reviews have indicated an association between sedentary behavior (leisure-time sitting, television viewing, total and occupational sitting time) and cancer-related mortality, [30], [40] others have found no such association. [31], [33] However, the latter evaluated the total number of deaths from cancer regardless of the etiology.

#### Cardiovascular disease

Five systematic reviews investigated the association between sedentary behavior and cardiovascular disease in adults. [29], [33], [34], [37], [40] Two of these reviews indicated that there are conflicting results regarding sedentary behavior (occupational and general), in terms of cardiovascular outcomes, [33], [40] underscoring the fact that there have been few studies addressing this topic. More recently, two systematic reviews that included meta-analyses concluded that the results are consistent and show a significant positive association between sedentary behavior (≥2 television hours/day; screen-time and sitting time) and cardiovascular disease, regardless of the level of physical activity, with summary measures of 1.15 (95% CI, 1.06–1.23) and 2.47 (95% CI, 1.44–4.24), respectively. [29], [34] In addition, in the most recent meta-analysis, Ford et al., [37] found that 2 hours/day of screen- time and sitting time were associated with an increase of 5% (HR 1.05; 95% CI 1.01–1.09) and 17% (HR 1.17; 95% CI 1.13–1.20) in cardiovascular events, respectively.

#### Cancer

Five systematic reviews investigated the association between sedentary behavior and cancer in adults. [30], [31], [33], [35], [40] These reviews showed that sedentary behavior (overall sitting time, sitting outside of work, and TV viewing) is associated with an increase in the risk of colorectal, [33], [35], [40] breast, [30], [40] endometrial, [31], [33] ovary, [33], [40] and prostate cancer. [30] However, conclusions are still based on a limited number of studies, some of which did not consider confounding factors such as BMI and physical activity. [31], [33] Additionally, van Uffelen et al. [40] stated that there is no established association between occupational sitting time and renal, prostate, lung or testicular cancer.

#### Type 2 diabetes

Five systematic reviews concluded that there is a significant and positive association between sedentary behavior and type 2 diabetes in adults, regardless of physical activity level. [29], [31], [33], [34], [40] The meta-analysis conducted by Grøntved and Hu [29] found that watching television for more than 2 h per day was associated with a 20% increase in the risk of type 2 diabetes (RR, 1.20; 95% CI, 1.14–1.27). According to Wilmot et al., [34] adults that spend most of their time engaged in sedentary behavior (screen-time and sitting time), compared to adults who spend very little time engaged in such behavior, are at increased risk of developing type 2 diabetes (RR, 2.12; 95% CI 1.61, 2.78). However, these authors included 5 cross-sectional studies and 5 prospective studies for the summary measure in their meta-analysis. When the meta-analysis included only the prospective studies, the results, although still statistically significant, were attenuated.

#### Metabolic syndrome and individual cardiovascular risk factors

One systematic review evaluated the association between sedentary behavior and metabolic syndrome; [28] four evaluated the association between sedentary behavior and individual cardiovascular risk factors, [15], [18], [31], [33] and one evaluated both associations.[26].

In children and adolescents, two reviews have been published. [15], [26] For Chinapaw et al., [15] there is insufficient evidence for a longitudinal relationship between sedentary time and blood pressure or blood lipids. In contrast, Tremblay et al. [6] reported that there is longitudinal evidence (studies with moderate quality) linking sedentary behavior (television, screen-time, and self-reported sedentary behavior) with total cholesterol and blood pressure; however, there was insufficient evidence for metabolic syndrome.

In adults, time spent in sedentary behavior (television viewing and screen-time) is associated with metabolic syndrome, regardless of the level of physical activity. [28] However, the evidence is insufficient for individual cardiovascular risk factors (e.g., blood pressure, blood lipids and cholesterol levels). [31], [33].

#### Obesity, overweight and adiposity

Fourteen systematic reviews examined whether sedentary behavior was associated with body mass index, weight gain, overweight/obesity and adiposity in children, adolescents[15]–[19], [22]–[27] and adults. [31], [33], [40].

In a meta-analysis including randomized controlled trails, Tremblay et al. [26] concluded that TV viewing in children and adolescents leads to obesity. Similar conclusions were reported by the following reviews: Marshall et al., [19] Rey-Lopez et al., [23] Prentice-Dunn et al., [22] Costigan et al., [16] Hoare et al., [17] and Salmon et al.,[25] despite their lower methodological quality. In preschool children (4–6 years), [27] there was moderate evidence for an association between TV viewing and overweight. Similarly, Leblanc et al. [18] found low- to moderate- quality evidence linking TV viewing with unfavorable measures of adiposity. Finally, according to Chinapaw et al. [15] insufficient evidence for a longitudinal positive relationship between ‘sedentary time’ – mainly TV viewing – and adiposity exist. The obesogenic effect of sedentary behavior may be mediated by unhealthy dietary behaviors [21], [24] and lower physical activity levels. [24].

In contrast, in adults, the obesogenic effect of sedentary behavior is not supported by observational studies. For Thorp et al. [33] limited evidence for a longitudinal relationship exists between sedentary behavior, weight gain, and risk of obesity. Similarly, insufficient evidence was concluded for body weight–related measures in Proper et al. [31] Finally, in van Uffelen et al., “prospective studies failed to confırm a causal relationship” [40].

#### Mental health

Few systematic reviews have examined whether sedentary behavior is associated with mental disorders in children, [16], [17], [26] and with mental [33] and depressive disorders in adults. [31], [32], [39].

In children and adolescents, sedentary behavior (screen time) was associated with depression; however, evidence was based on cross-sectional studies. [16], [17], [26] In adults, some reviews reported an association between sedentary behavior (television and other sedentary behaviors) and depressive symptoms [32], [33] and postnatal depressive symptoms, [39] also based on cross-sectional studies.

#### Musculoskeletal disorders

Four systematic reviews investigated the association between sedentary behavior and musculoskeletal disorders. [16], [36], [38], [41] In children, there is insufficient evidence on the association between exposure to screen-based sedentary behavior and musculoskeletal [16] and low back pain. [36] Similarly, for adults, there is limited evidence on the association between sedentary behavior (occupational sitting; computer use; sedentary behavior and prolonged sitting-time during leisure; and total sitting time) and low back pain, neck pain, shoulder pain, hand pain and arm pain. [36], [38], [41].

#### Other Behaviors

Other systematic reviews analyzed whether sedentary behavior was associated with: physical activity, [19], [24] aggression, [20] unhealthy dietary intake (e.g., between meal snacks, sweets and beverages), [21], [24], [25] and pro-social behaviors [26] in children. In adults, smoking,[25] less leisure time physical activity, [19], [24] alcohol consumption and eating have all been examined in relation to sedentary behavior.[25].

In children with emotional and environmental difficulties, there is insufficient evidence for the association between television viewing and aggressiveness. [20] Studies of children, in general, have demonstrated a significant inverse relationship, albeit a weak one, between television viewing and engaging in physical activity. [19], [24] In such studies, television viewing was associated with lower self-esteem and pro-social behaviors.[26] In addition, Pearson and Biddle [21] conducted a review in which they concluded that sedentary behavior, predominantly screen time, is associated with unhealthy eating habits (alcohol consumption and eating behavior in adults) in adults, adolescents, and children, although most of the articles evaluated were cross-sectional studies.

#### Other outcomes

Systematic reviews have investigated the association between sedentary behavior and other health outcomes, such as bone health, [15], [26] psychosocial and motor dysfunction, [18], [26] poor academic performance and cognitive development, [18], [26] physical fitness, [15], [26] and symptomatic gallstone disease. [33].

Two recent systematic reviews found a significant inverse association between television viewing time and academic performance, as measured by IQ, grades/grade point average, and performance on standardized tests.[18], [26] Television viewing was also associated with low cognitive performance, worse reading comprehension, low math scores, less classroom engagement, worse comprehension, low memory, reduced attention and number of vocalizations, and language delay. [18].

Other studies showed that, in children, increased television viewing time was associated with poor psychosocial health (e.g., poor social behaviors and low self-esteem). [18], [25] Studies with moderate quality indicated an association between sedentary behavior (TV watching and playing computer games) and physical fitness (including general physical fitness, aerobic power and neuromotor) in children and youth, [15], [26] regardless of the physical activity level.

There is insufficient data to draw any conclusions regarding the relationship between sedentary behavior and bone mineral density [15], [26] in children and symptomatic gallstone disease in adults.[33].

### Use of Causative Language

Of the 27 reviews included in this overview, 24 (89%) made a correct use of causative language taking into account the design of the included studies (File S6). Among all the systematic reviews, only Tremblay et al. [26] included randomized controlled trials, and therefore, use a proper qualified causal language describing their own conclusions. On the other hand, three systematic reviews [17], [19], [37] provided greater inferential strength than their review warranted. Taken together, most of the researchers were aware of the limitations presented in each review to establish the best scientific evidence.

## Overall Conclusions

The present overview summarizes the current knowledge about the role of sedentary behavior on human health. The main limitations of this overview were that we drew conclusions based only on systematic reviews of observational studies. Unfortunately, we identified very few systematic reviews of RCTs in our initial research strategy. In addition, the main focus of the RCT reviews were only to analyze the efficacy of interventions to reduce sedentary behavior and/or the effect on short-term health outcomes. [48], [49].

It is important to highlight that the observational evidence between sedentary behavior and different health outcomes reported in this overview is complex, depending on the type of sedentary behavior and the age groups studied (File S5).

In children and adolescents, there is strong evidence of a relationship between sedentary behavior (based on TV viewing and screen-time) and obesity. Moreover, we found moderate evidence for blood pressure and total cholesterol, self-esteem, social behavior problems, physical fitness and academic achievement (based on TV viewing and screen-time).

In adults, we found strong evidence of a relationship between sedentary behavior and all-cause mortality, fatal and non-fatal cardiovascular disease (based on TV viewing, screen-time and sitting time), type 2 diabetes (TV viewing and screen-time) and metabolic syndrome (based on TV viewing, screen-time, sitting time and objectively measured sedentary time). In addition, there is moderate evidence for incidence rates of ovarian (sitting time), colon (TV viewing) and endometrial cancers (sitting outside of work and overall sitting) and type 2 diabetes (sitting time).

Finally, there is inconclusive evidence for certain health outcomes in adults (e.g., cancer mortality, incidence of breast cancer, colorectal cancer and ovarian cancer, individual cardiovascular risk factors, depressive symptoms, musculoskeletal disorders, health behaviors symptomatic gallstone disease), children and adolescents (e.g., metabolic syndrome, some individual cardiovascular, mental health, musculoskeletal, other health behaviors, bone mass, motor dysfunction), and thus, further studies are needed.

## Perspectives

### Future directions for original studies

Reviews included a predominance of cross-sectional studies, which do not allow us to infer causality between variables. Although prospective studies with high methodological quality may provide better insight of the role of sedentary behavior on human health, residual confounding may still exist. Ideally, randomized controlled trials should be conducted to confirm deleterious effects attributed to some sedentary behaviors. However, a high-quality randomized controlled trial designed to analyze the effect of sedentary behavior on endpoint health outcomes may be unfeasible because of its high cost and reduced compliance with the intervention. Nonetheless, other epidemiologic study designs (Mendelian randomization, [50] twin studies [51] and high quality observational longitudinal studies with at least three observations for exposure and outcome per individual) [52] may also provide a stronger evidence of causality.

Future epidemiological studies should not employ just one or few sedentary behaviors as an overall marker of sedentary behavior, [53] because there is growing epidemiological evidence that certain sedentary activities are more detrimental for health than others. For example, it is well established that the adverse health effects attributed to TV viewing may be mediated by unhealthy dietary patterns [21], which is less common in people who spend more time in other sedentary behaviours (such as reading, studying).

Consequently, to increase the current knowledge of sedentary behavior, future studies must incorporate emergent objective methods (i.e., geolocation data combined with acceleration signals in mobile phones, small video cameras, and inclinometers) to obtain an accurate measure and contextual information of sedentary behavior. [54] Including more accurate tools to evaluate sedentary behavior may be an important advance in sedentary behavior studies because it may enhance the magnitude of the observed associations. [55] Further information regarding methods of measurement in epidemiological studies of sedentary behavior have been described in detail elsewhere. [54] Finally, the evaluation of confounders (i.e., physical activity) should also receive special attention; otherwise, residual confounding may still be present.

### Future directions for systematic reviews

From our point of view, future systematic reviews should assess the quality of the original articles and make conclusions based on it. Other AMSTAR items that were infrequently performed/reported and merit future considerations were: prior design of the systematic review, the use of two independent reviewers and data extractors, inclusion of grey literature, list of included and excluded articles selected, evaluation of heterogeneity and publication bias. Systematic reviews of observational studies should also detail how confounding variables (i.e., physical activity) were assessed in each original study. In addition, the main limitations involving meta-analysis of observational studies (i.e., different measurements of exposure and outcomes, heterogeneity, confounding and bias between studies) should be rigorously considered. [56].

Finally, no systematic reviews have been exclusively performed in elderly individuals. Therefore, we encourage future investigations of sedentary behavior in this age group because they spend most of their daily time in sedentary activities. [12], [13].

## Supporting Information

File S1 **Search strategy.**
(DOCX)

File S2 **Characteristics of the systematic reviews examining the relationship between sedentary behavior and health outcomes.**
(DOC)

File S3 **Eligibility criteria and extraction of physical activity adjustment within the systematic reviews.**
(DOC)

File S4 **Methodological quality assessment of systematic reviews.**
(DOCX)

File S5 **Level of scientific evidence for associations between sedentary behaviors and health outcomes, by age group and type of sedentary behavior.**
(DOCX)

File S6 **Aims, main results and use of causative language in systematic reviews of sedentary behavior and health outcomes.**
(DOCX)

Checklist S1 **PRISMA Checklist.**
(DOC)
