# Supplementary material for: Sedentary Behavior and Health Outcomes: An Overview of Systematic Reviews
Source: PLoS One. 2014 Aug 21;9(8):e105620. doi: 10.1371/journal.pone.0105620 (PMC4140795; doi:10.1371/journal.pone.0105620)
Supplement: File S1 — Search strategy. (DOCX) [file pone.0105620.s001.docx]

**SUPPLEMENTARY FILE 1**

**SEARCH STRATEGY**

**Search strategy in 02^nd^ September 2013**

**Total – 1044**

**Removing Duplicate articles - 893**

**Medline – 424 articles**

((((physical inactivity)) OR (physical activity))) AND ((((((((((((((sedentary behavior)) OR (sedentary lifestyles)) OR (sedentary time)) OR (sitting time)) OR (tv viewing)) OR (television viewing)) OR (tv watching)) OR (television watching)) OR (driving)) OR ("screen based")) OR (video game)) OR (computer)) OR ("screen time")) Filters activated: Systematic Reviews, Meta-Analysis

**Detailed search**

((("physical examination"[MeSH Terms] OR ("physical"[All Fields] AND "examination"[All Fields]) OR "physical examination"[All Fields] OR "physical"[All Fields]) AND inactivity[All Fields]) OR ("motor activity"[MeSH Terms] OR ("motor"[All Fields] AND "activity"[All Fields]) OR "motor activity"[All Fields] OR ("physical"[All Fields] AND "activity"[All Fields]) OR "physical activity"[All Fields])) AND (((((((((((((sedentary[All Fields] AND ("behaviour"[All Fields] OR "behavior"[MeSH Terms] OR "behavior"[All Fields])) OR ("sedentary lifestyle"[MeSH Terms] OR ("sedentary"[All Fields] AND "lifestyle"[All Fields]) OR "sedentary lifestyle"[All Fields] OR ("sedentary"[All Fields] AND "lifestyles"[All Fields]) OR "sedentary lifestyles"[All Fields])) OR (sedentary[All Fields] AND ("time"[MeSH Terms] OR "time"[All Fields]))) OR (sitting[All Fields] AND ("time"[MeSH Terms] OR "time"[All Fields]))) OR (tv[All Fields] AND viewing[All Fields])) OR (("television"[MeSH Terms] OR "television"[All Fields]) AND viewing[All Fields])) OR (tv[All Fields] AND watching[All Fields])) OR (("television"[MeSH Terms] OR "television"[All Fields]) AND watching[All Fields])) OR ("automobile driving"[MeSH Terms] OR ("automobile"[All Fields] AND "driving"[All Fields]) OR "automobile driving"[All Fields] OR "driving"[All Fields])) OR "screen based"[All Fields]) OR ("video games"[MeSH Terms] OR ("video"[All Fields] AND "games"[All Fields]) OR "video games"[All Fields] OR ("video"[All Fields] AND "game"[All Fields]) OR "video game"[All Fields])) OR ("computers"[MeSH Terms] OR "computers"[All Fields] OR "computer"[All Fields])) OR "screen time"[All Fields])

**Psychinfo – 39 articles**

edentary behavior OR sedentary lifestyles OR sedentary time OR sitting time OR tv viewing OR television viewing OR tv watching OR television watching OR driving OR "screen based" OR video game OR computer OR "screen time""

**web of science – 333 articles**

(physical inactivity OR physical activity) AND (sedentary behavior OR sedentary lifestyles OR sedentary time OR sitting time OR tv viewing OR television viewing OR tv watching OR television watching OR driving OR "screen based" OR video game OR computer OR "screen time") AND (systematic review OR meta-analysis OR evidence-base medicine)

**embase – 248 articles**

**
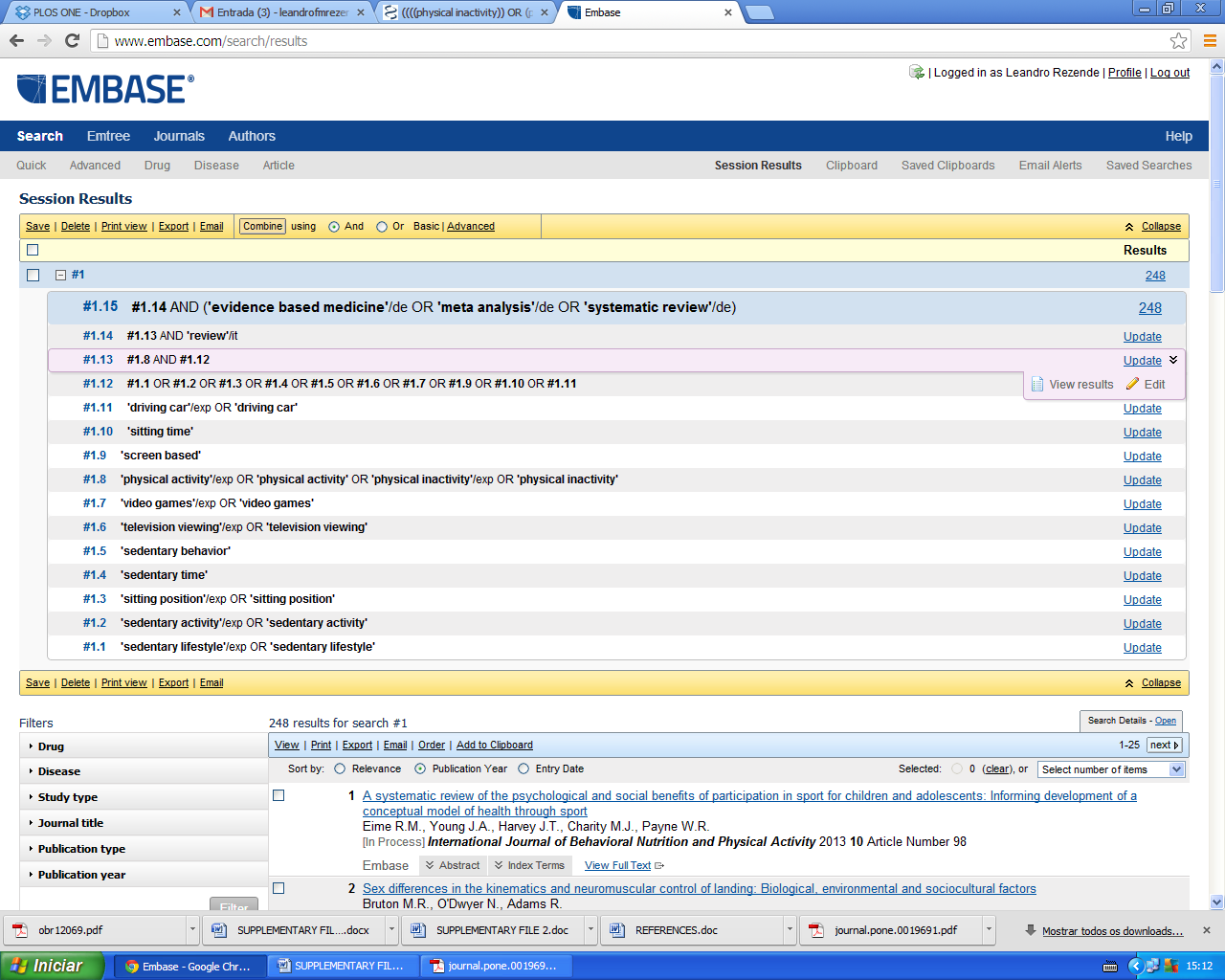
**
