# Supplementary material for: Sedentary Behavior and Health Outcomes: An Overview of Systematic Reviews
Source: PLoS One. 2014 Aug 21;9(8):e105620. doi: 10.1371/journal.pone.0105620 (PMC4140795; doi:10.1371/journal.pone.0105620)
Supplement: File S2 — Characteristics of the systematic reviews examining the relationship between sedentary behavior and health outcomes. (DOC) [file pone.0105620.s002.doc]

**SUPPLEMENTARY FILE 2** Characteristics of the systematic reviews examining the relationship between sedentary behaviour and health outcomes.

| **Author** | **Year** | **Age group** | **Type of Sedentary behaviour‡** | **Outcome measure** | **Meta-analysis** | **Quality assessment** |
| --- | --- | --- | --- | --- | --- | --- |
| **Children and Adolescents** | |  |  |  |  |  |
| Chinapaw *et al^15^* | 2011 | < 18 years | no restriction | Biomedical health indicators | No | Yes |
| Costigan *et al^16^* | 2013 | 12-18 years | Screen-based sedentary behavior | Health Indicator (Physical, psychosocial, and/or behavioural) | No | Yes |
| Hoare *et al^17^* | 2013 | 10-19 years | no restriction | Depression | No | No |
| LeBlanc *et al^18^* | 2012 | 0–4 years | no restriction | Adiposity, bone mass, motor development, psychosocial health, cognitive development, cardiometabolic health | No | Yes |
| Marshall *et al^19^* | 2004 | 3–18 years | Television viewing, video/computer game use | Increased body fat and physical activity | Yes | No |
| Mitrofan *et al^20^* | 2008 | < 18 years | Television viewing and videogame playing | Childhood aggression | No | Yes |
| Pearson and Biddle^21^ | 2011 | < 11 years; 12–18 years; and > 18 years | no restriction | Dietary intake | No | Yes |
| Prentice-Dunn and Prentice-Dunn^22^ | 2012 | 2–19 years | no restriction | Obesity | No | No |
| Rey-López et al^23^ | 2008 | 2-18 years | no restriction | Overweight and obesity | No | No |
| Rossi *et al^24^* | 2010 | 6–19 years | Television viewing | Dietary intake and obesity | No | No |
| Salmon *et al^25^* | 2011 | 0-18 years | no restriction | Obesity, unhealthy diet, tobacco use, drug, and alcohol use, socio-cognitive outcomes, between meal snacking, depression. | No | No |
| Tremblay *et al^26^* | 2012 | 5–17 years | no restriction | Body composition, physical fitness, metabolic syndrome, cardiovascular risk, self-esteem, pro-social behaviour, academic performance | Yes | Yes |
| Velde *et al^27^* | 2012 | 4–6 years | no restriction | Obesity | No | Yes |
| **Adults** |  |  |  |  |  |  |
| Edwardson *et al^28^* | 2012 | > 18 years | no restriction | Metabolic syndrome | Yes | Yes |
| Grontved and Hu^29^ | 2011 | > 18 years | Television viewing | Type 2 diabetes, (fatal or non-fatal) cardiovascular disease, and all-cause mortality | Yes | No |
| Lynch *et al^30^* | 2010 | > 18 years | no restriction | Colorectal, endometrial, ovarian, and prostate cancer risk; cancer mortality; and weight gain | No | No |
| Pearson and Biddle^21^ | 2011 | < 11 years; 12–18 years; and > 18 years | no restriction | Dietary intake | No | Yes |
| Proper *et al^31^* | 2011 | > 18 years | no restriction | Increased BMI, obesity, increased waist circumference, mortality, type 2 diabetes, cardiovascular risk factors, and endometrial cancer | No | Yes |
| Teychenne *et al^32^* | 2010 | 18-60 | no restriction | Depression | No | Yes |
| Thorp *et al^33^* | 2011 | > 18 years | no restriction | Mortality, disease incidence, obesity, weight gain, cardiometabolic biomarkers | No | No |
| Wilmot *et al^34^* | 2012 | > 18 years | no restriction | Type 2 diabetes, cardiovascular disease, and all-cause mortality | Yes | Yes |
| **Unspecified ages** |  |  |  |  |  |  |
| Boyle^35^ | 2012 | Unspecified age group | no restriction | Colon cancer | No | No |
| Chen *et al^36^* | 2009 | Unspecified age group | Prolonged sitting at work and during leisure time | Low back pain | No | Yes |
| Ford and Caspersen^37^ | 2012 | Unspecified age group | Screen-time and sitting time | Fatal and non-fatal Cardiovascular disease | Yes | No |
| IJmker *et al^38^* | 2007 | Unspecified age group | Occupational computer use | Hand-arm and neck-shoulder symptoms and disorders | No | Yes |
| Teychenne et al.^39^ | 2013 | Unspecified age group | no restriction | Postnatal Depressive Symptoms | No | Yes |
| van Uffelen *et al^40^* | 2010 | Unspecified age group | Occupational sitting | BMI, cancer, cardiovascular disease, type 2 diabetes, and mortality | No | Yes |
| Waersted *et al^41^* | 2010 | Unspecified age group | Computer work | Neck and upper extremity disorders (except carpal tunnel syndrome) | No | Yes |

‡For review articles evaluating unspecified sedentary behaviours, all sedentary behaviours were collectively defined as "no restriction".
