# Supplementary material for: Sedentary Behavior and Health Outcomes: An Overview of Systematic Reviews
Source: PLoS One. 2014 Aug 21;9(8):e105620. doi: 10.1371/journal.pone.0105620 (PMC4140795; doi:10.1371/journal.pone.0105620)
Supplement: File S3 — Eligibility criteria and extraction of physical activity adjustment within the systematic reviews. (DOC) [file pone.0105620.s003.doc]

**SUPPLEMENTARY FILE 3** Eligibility criteria and extraction of physical activity adjustment within the systematic reviews.

| **Author** | **Inclusion Criteria** | | | **Exclusion Criteria** | | | | **Extraction Physical Activity as an Adjustment** | **How Many Studies Adjusted How an Physical Activity was Assessed** |  |
| --- | --- | --- | --- | --- | --- | --- | --- | --- | --- | --- |
| **Children and Adolescents** | |  |  | |  |  |  | | | |
| Chinapaw *et al^15^* | - Prospective studies – relationship between sedentary behaviour and health indicators.  - Full-text articles published in English | | | N/A | | | | Yes | Yes - (14/31) = 45%  Questionnaire: N/A  Objectively: N/A |  |
| Costigan *et al^16^* | - Adolescent girls aged 12 e 18 years  - Examined in adolescent girls or separately by gender  - Cross-sectional, longitudinal, or experimental study design  - Assessed leisure-time screen- based sedentary behavior and at least one health indicator | | | - Conference abstracts, dissertations, theses and articles in non-peer-reviewed journals - Screen-time in an educational context and; considered the content viewed - Active electronic gaming | | | | Yes | Yes - (16/33) = 49%  Questionnaire: N/A  Objectively: N/A |  |
| Hoare *et al^17^* | - Young people between 10–19 years | | | - Particular groups (e.g. disability) and/or pilot or feasibility studies | | | | N/A | N/A |  |
| LeBlanc *et al^18^* | - Sedentary behaviour time and health indicator in the early years (infants: 1 month – 1.0 years; toddlers: 1.1–3.0 years; preschoolers: 3.1– 4.99 years)  - RCT, quasi-experimental, intervention,  prospective cohort, or any study that has either a comparison group or a follow-up period | | | - Papers published in other languages than English or French - Active electronic gaming | | | | N/A | N/A |  |
| Marshall *et al^19^* | - Participants less than 18 y of age  - Published in English in peer-reviewed journals | | | N/A | | | | N/A | N/A |  |
| Mitrofan *et al^20^* | - Quantitative and qualitative studies  - Associations between aggressive content and amount of television viewing and video game playing and aggression in children and young people with behavioural and emotional difﬁculties | | | - Studies examining aggression-related phenomena (e.g. thoughts, feelings or mood) | | | | N/A | N/A |  |
| Pearson and Biddle^21^ | - Children <11 years; Adolescents 12-18 years; >18 years  - At least one measure of sedentary behavior  - Observational studies  - Published up to January 2010 | | | - Experimental Studies | | | | N/A | N/A |  |
| Prentice-Dunn and Prentice-Dunn^22^ | - Cross-sectional studies published between 2000–2010  - Children: ages 2–19 | | | - Fewer than 100 subjects were excluded | | | | N/A | N/A |  |
| Rey-López et al^23^ | - Children between 2-18 years old  - Published between 1990-2007 | | | - N/A | | | | N/A | N/A |  |
| Rossi *et al^24^* | - Open-access full-published articles  - Published in English and Portuguese.  - Children between 6 to 19 years  - Published between 2000-2010. | | | - Communications, reviews, and particular groups (e.g. disability) | | | | N/A | N/A |  |
| Salmon *et al^25^* | - Children and adolescents: ages 0–18 | | | - N/A | | | | N/A | N/A |  |
| Tremblay *et al^26^* | - Children and adolescents: ages 5–18  - All study designs | | | - Population based studies a minimum sample size of 300 participants; RCTs, and intervention with at least 30 participants.  - Active electronic gaming  - Sedentary behaviour as ‘failing to meet physical activity guidelines’ | | | | N/A | N/A |  |
| Velde *et al^27^* | - Prospective studies  - Children aged 4–6 years at baseline  - Anthropometric measurements  - Only full-text papers published in English were included. | | | - Studies addressing nutrition disorders,  - Reporting parental behaviours (e.g. feeding practices)  - Studies including a selected group of children | | | | N/A | N/A |  |
| **Adults** |  | | |  | | | |  |  |  |
| Edwardson *et al^28^* | - Cross sectional or prospective design;  - Adults >18 years of age;  - Self-report or objective measure of time spent sedentary;  - Include an outcome measure of metabolic syndrome; and (5)  - Published in English | | | - Sedentary behaviour as ‘failing to meet physical activity guidelines’ | | | | Yes | Yes - (8/10) = 80%  Questionnaire: 7 studies  Objectively: 1 study |  |
| Grontved and Hu^29^ | - Published in the English  - Prospective design (cohort, case-cohort, and nested case-control)  - Population that was healthy at baseline  - Had estimates of relative risk (RR) or OR with 95% CIs | | | - N/A | | | | Yes | Yes - (7/8) = 88%  Questionnaire: N/A  Objectively: N/A |  |
| Lynch *et al^30^* | - Written in English between 1980 – June 2010  - Non-pregnant adults (  - Sedentary behavior as an independent variable from physical activity | | | - Sedentary behaviour as ‘failing to meet physical activity guidelines’ | | | | Yes | Yes - (14/18) = 78%  Questionnaire: N/A  Objectively: N/A |  |
| Pearson and Biddle^21^ | - Children <11 years; Adolescents 12-18 years; >18 years  - At least one measure of sedentary behavior  - Observational studies  - Published up to January 2010 | | | - Experimental Studies | | | | N/A | N/A |  |
| Proper *et al^31^* | - Prospective Design  - Healthy population>18 years | | | N/A | | | | Yes | Yes - (12/19) = 63%  Questionnaire: N/A  Objectively: N/A |  |
| Teychenne *et al^32^* | - Both observational and experimental  - Healthy adults – 18 – 60 years  - Valid indicator risk of depression | | | Abstract and dissertations | | | | N/A | N/A |  |
| Thorp *et al^33^* | - Longitudinal Studies  - Adults >18 years  - Sedentary behavior and health outcomes | | | N/A | | | | Yes | Yes - (34/48) = 71%  Questionnaire: N/A  Objectively: N/A |  |
| Wilmot *et al^34^* | - Cross-sectional or prospective design;  - Adults ≥18 years of age;  - Self- reported or objective measure of time spent sedentary;  - Report data on a relevant health outcome | | | - Sedentary behaviour as ‘failing to meet physical activity guidelines’ | | | | Yes | Yes - (10/18) = 56%  Questionnaire: N/A  Objectively: N/A |  |
| **Unspecified ages** |  | | |  | | | |  |  |  |
| Boyle^35^ | Relationship Sedentary behavior and Cancer | | | N/A | | | | N/A | N/A |  |
| Chen *et al^36^* | - Sitting and low back pain studies  - Cohort or Case-control | | | - Specific spinal condition | | | | N/A | N/A |  |
| Ford and Caspersen^37^ | - Sedentary behavior and mortality  - Adults  Publications written in English | | |  | | | | Yes | Yes - (11/11) = 100%  Questionnaire: 11  Objectively: 0 |  |
| IJmker *et al^38^* | - Study population computer workers and syndromes, signs or symptoms related to pain or discomfort in hand, arm, shoulder or neck;  - A risk estimate of the association  - Longitudinal study  - Full-text, peer-reviewed article, written in English, Dutch or German | | | Experimental studies, abstracts and letters | | | | N/A | N/A |  |
| Teychenne et al.^39^ | - Observational study | | | - Dissertations and abstracts | | | | N/A | N/A |  |
| van Uffelen *et al^40^* | - Adults  - Occupational sitting time and the relationship with health outcomes  - Only full-texts | | | N/A | | | | Yes | Yes - (19/43) = 44%  Questionnaire: N/A  Objectively: N/A |  |
| Waersted *et al^41^* | - Working age population  - computer sitting time and the relationship with musculoskeletal disorders (objective examination)  - Only full-texts | | | - Effects of intervention on musculoskeletal disorders related to computer use | | | | Yes | Yes - (4/22) = 15%  Questionnaire: N/A  Objectively: N/A |  |
