# Supplementary material for: Sedentary Behavior and Health Outcomes: An Overview of Systematic Reviews
Source: PLoS One. 2014 Aug 21;9(8):e105620. doi: 10.1371/journal.pone.0105620 (PMC4140795; doi:10.1371/journal.pone.0105620)
Supplement: File S4 — Methodological quality assessment of systematic reviews. (DOCX) [file pone.0105620.s004.docx]

**SUPPLEMENTARY FILE 4:** Methodological quality assessment of systematic reviews

|  |  | **Methodological Quality Assessment of the included studies Systematic Reviews - AMSTAR Itens** | | | | | | | | | | | |
| --- | --- | --- | --- | --- | --- | --- | --- | --- | --- | --- | --- | --- | --- |
|  |  | **1** | **2** | **3** | **4** | **5** | **6** | **7** | **8** | **9** | **10** | **11** | **Rating** |
| **Children and Adolescents** | | | | | | | | | | | | | |
| Chinapaw et al^15^ | | Yes | Yes | Yes | No | No | Yes | Yes | Yes | N/A | No | Yes | 7 |
| Costigan et al^16^ | | Yes | No | Yes | No | No | Yes | Yes | Yes | N/A | No | Yes | 6 |
| Hoare et al^17^ | | Yes | No | No | No | Yes | Yes | No | No | N/A | No | Yes | 4 |
| LeBlanc et al^18^ | | Yes | Yes | Yes | No | No | Yes | Yes | Yes | Yes | No | Yes | 8 |
| Marshall et al^19^ | | Yes | No | Yes | No | No | No | No | No | Yes | No | No | 3 |
| Mitrofan et al^20^ | | Yes | Yes | Yes | Yes | No | Yes | Yes | Yes | N/A | No | No | 7 |
| Pearson and Biddle^21^ | | Yes | No | Yes | No | No | Yes | Yes | Yes | N/A | No | Yes | 5 |
| Prentice-Dunn and Prentice-Dunn^22^ | | Yes | No | No | No | No | Yes | No | No | N/A | No | No | 2 |
| Rey-López et al^23^ | | Yes | No | No | No | No | Yes | No | No | N/A | No | Yes | 3 |
| Rossi et al^24^ | | Yes | No | Yes | No | No | Yes | No | No | N/A | No | No | 3 |
| Salmon et al^25^ | | Yes | No | Yes | No | No | No | No | No | N/A | No | Yes | 3 |
| Tremblay et al^26^ | | Yes | Yes | Yes | Yes | No | Yes | Yes | Yes | Yes | Yes | Yes | 10 |
| Velde et al^27^ | | Yes | Yes | Yes | No | No | Yes | Yes | Yes | N/A | No | Yes | 7 |
| **Adults** | | | | | | | | | | | | | |
| Edwardson et al^28^ | | Yes | Yes | Yes | No | No | Yes | Yes | Yes | Yes | Yes | Yes | 9 |
| Grontved and Hu^29^ | | Yes | No | Yes | No | No | Yes | No | No | Yes | Yes | Yes | 6 |
| Lynch et al^30^ | | Yes | No | Yes | No | No | Yes | No | No | N/A | No | Yes | 4 |
| Pearson and Biddle^21^ | | Yes | No | Yes | No | No | Yes | Yes | Yes | N/A | No | Yes | 5 |
| Proper et al^31^ | | Yes | No | Yes | No | No | Yes | Yes | Yes | N/A | No | Yes | 6 |
| Teychenne et al^32^ | | Yes | No | Yes | No | No | Yes | Yes | Yes | N/A | No | Yes | 6 |
| Thorp et al^33^ | | Yes | No | Yes | No | No | Yes | No | No | N/A | No | Yes | 4 |
| Wilmot et al^34^ | | Yes | Yes | Yes | No | No | Yes | Yes | Yes | Yes | Yes | Yes | 9 |
| **Unspecified ages** | |  |  |  |  |  |  |  |  |  |  |  |  |
| Boyle^35^ | | Yes | No | Yes | No | No | Yes | No | No | N/A | No | No | 3 |
| Chen et al^36^ | | Yes | No | Yes | No | No | No | Yes | Yes | N/A | Yes | No | 5 |
| Ford and Caspersen^37^ | | Yes | No | No | No | No | Yes | No | No | Yes | No | Yes | 4 |
| IJmker et al^38^ | | Yes | Yes | Yes | No | No | Yes | Yes | Yes | N/A | No | Yes | 7 |
| Teychenne et al.^39^ | | Yes | No | Yes | No | No | Yes | Yes | Yes | N/A | No | Yes | 6 |
| van Uffelen et al^40^ | | Yes | No | Yes | No | No | No | Yes | Yes | N/A | No | Yes | 5 |
| Waersted et al^41^ | | Yes | No | Yes | No | No | Yes | Yes | Yes | N/A | No | Yes | 6 |
| **TOTAL** | | 27 | 8 | 23 | 2 | 2 | 23 | 16 | 16 | 7 | 5 | 21 |  |
| **%** | | 100 | 30% | 85% | 7% | 7% | 85% | 59% | 59% | 26% | 18% | 28% |  |

AMSTAR contains 11-items to appraise the methodological aspects of the systematic reviews. All 11-items were scored as “Yes”, “No”, “Can’t Answer” or “Not Applicable”. AMSTAR comprises the following items:

1. ‘a priori’ design provided;

2. duplicate study selection/data extraction;

3. comprehensive literature search;

4. status of publication as inclusion criteria (i.e., grey or unpublished literature);

5. list of studies included/excluded provided;

6. characteristics of included studies documented;

7. scientific quality assessed and documented;

8. appropriate formulation of conclusions (based on methodological rigor and scientific quality of the studies);

9. appropriate methods of combining studies (homogeneity test, effect model used and sensitivity analysis);

10. assessment of publication bias (graphic and/or statistical test); and

11. conflict of interest statement.
