# Supplementary material for: Sedentary Behavior and Health Outcomes: An Overview of Systematic Reviews
Source: PLoS One. 2014 Aug 21;9(8):e105620. doi: 10.1371/journal.pone.0105620 (PMC4140795; doi:10.1371/journal.pone.0105620)
Supplement: File S5 — Level of scientific evidence for associations between sedentary behaviors and health outcomes, by age group and type of sedentary behavior. (DOCX) [file pone.0105620.s005.docx]

|  |  |  |
| --- | --- | --- |
|  |  |  |
|  |  |  |
| **OUTCOMES** | **CHILDREN/ADOLESCENTS** | **ADULTS** |
| **Mortality** |  |  |
| All-cause mortality | No evidence | Strong Evidence^1,2,3^ |
| CVD mortality | No evidence | Strong Evidence^1,2,3^ |
| Cancer Mortality | No evidence | No Evidence^1,3,4^ |
| **Cardiovascular Diseases** | No evidence | Strong Evidence^1,2,3^  Insufficient Evidence^4^ |
| **Cancer** |  |  |
| Breast | No evidence | Insufficient Evidence^4^ |
| Colorectal | No evidence | Insufficient Evidence^4^ |
| Colon |  | Moderate Evidence^6^ |
| Endometrial | No evidence | Moderate Evidence^6^ |
| Ovarian | No evidence | Moderate Evidence^6^  Insufficient Evidence^4^ |
| Prostate | No evidence | Insufficient Evidence^6^ |
| **Type 2 Diabetes** | No evidence | Strong Evidence^1,2^  Moderate Evidence^3^  Insufficient Evidence^4^ |
| **Metabolic Syndrome** | Insufficient Evidence^1,2,3^ | Strong Evidence^1,2,3,5^ |
| **Individual Cardiovascular Risk Factors** |  |  |
| Blood Pressure | Moderate Evidence^1^ | Insufficient Evidence^1^ |
| Total Cholesterol | Moderate Evidence^1^ | Insufficient Evidence^1^ |
| HbA1 | Insufficient Evidence^1,2,3^ | Insufficient Evidence^1^ |
| Fasting Insulin | Insufficient Evidence^1,2,3^ | Insufficient Evidence^2^ |
| Insulin resistance | Insufficient Evidence^1,2,3^ | Insufficient Evidence^2^ |
| Leptin | No Evidence | Insufficient Evidence^1^ |
| Fibrinogen | No Evidence | Insufficient Evidence^1^ |
| C-peptide | No Evidence | Insufficient Evidence^1^ |
| **Obesity** | Strong Evidence^1,2^ | Insufficient Evidence^1,4^ |
| **Mental Health** |  |  |
| Self-Esteem | Moderate Evidence^1,2^ | No Evidence |
| Depressive Symptoms | No Evidence | Insufficient Evidence^3^ |
| Postnatal Depression | No Evidence | Insufficient Evidence^3,4^ |
| Cognitive Aspects | Insufficient Evidence^1^ | No Evidence |
| **Musculoskeletal** | Insufficient Evidence^2^ | Insufficient Evidence^4^ |
| **Other Behaviors (PA, diet, alcohol consumption)** | Insufficient Evidence^1^ | Insufficient Evidence^1^ |
| Social Behavior Problems | Moderate Evidence^1,2^ | No Evidence |
| **Other Health Outcomes** |  |  |
| Bone Mass | Insufficient Evidence^3^ | No Evidence |
| Motor Dysfunction | Insufficient Evidence^1^ | No Evidence |
| Physical Fitness | Moderate Evidence^1,2^ | No Evidence |
| Academic Achievement | Moderate Evidence^1,2^ | No Evidence |
| Symptomatic gallstone disease | No Evidence | Insufficient Evidence^5^ |

**1- Television viewing; 2- Screen-time; 3- Total Sitting time; 4- Occupational Sitting Time; 5- Objectively measured sedentary time; 6- Unspecified**

**All-Cause Mortality – conclusions based on Wilmot et al.**

**CVD Mortality – conclusions based on Wilmot et al.**

**Cancer Mortality – sitting time and TV was based on Proper; for occupational sitting time was based on van Uffelen**

**Grontved A, Hu FB (2011) Television viewing and risk of type 2 diabetes, cardiovascular disease, and all-cause mortality: a meta-analysis. JAMA 305: 2448-2455.**

**AMSTAR score - 6**

**Was scientific quality assessed and documented? No**

**Were conclusions formulated based on high methodological quality studies?** No

**Were the conclusions were based in longitudinal studies?** Yes

**Was physical activity extracted as covariate?** Yes - (7/8) = 88%

**Results/Conclusion**: “Our results from the meta-analysis of prospective cohort studies suggest that TV viewing is consistently associated with higher risk of type 2 diabetes, fatal or nonfatal cardiovascular disease, and all-cause mortality.”

**Level of Evidence - Moderate Evidence**

**Lynch BM (2010) Sedentary behavior and cancer: a systematic review of the literature and proposed biological mechanisms. Cancer Epidemiol Biomarkers Prev 19: 2691-2709.**

**AMSTAR score - 4**

**Was scientific quality assessed and documented? No**

**Were conclusions formulated based on high methodological quality studies?** No

**Were the conclusions were based in longitudinal studies?** Yes

**Was physical activity extracted as covariate?** Yes - (14/18) = 78%

**Results/Conclusion**: “Four prospective cohort studies examined the association between sedentary behavior (television in weekdays and weekends over the past 7 days, or the past year; total time spent sitting; total sitting outside of the work), and overall cancer mortality. One study observed a statistically significant increased risk (total sitting outside of the work), two studies observed nonsignificant risk increases (television in weekdays and weekends over the past 7 days, or the past year), and the fourth study presented null results (total time spent sitting).”

**Level of Evidence – Insufficient Evidence**

**Proper KI, Singh AS, van Mechelen W, Chinapaw MJM (2011) Sedentary behaviors and health outcomes among adults: A systematic review of prospective studies. Am J Prev Med. 40: 174-182.**

**AMSTAR score - 6**

**Was scientific quality assessed and documented? Yes**

**Were conclusions formulated based on high methodological quality studies?** Yes

**Were the conclusions were based in longitudinal studies?** Yes

**Was physical activity extracted as covariate?** Yes - (12/19) = 63%

**Results/Conclusion**: “Three studies investigated the relation ship between sedentary behavior and mortality. All three studies applied a different sedentary behaviour measures (occupational sitting, sitting time, and TV/videos viewing).” Among those studies, one was classified with low-quality (occupational sitting) and two with high quality. Based on the findings of the two high quality studies, there is a strong evidence for a relationship between sedentary behavior (total sitting time and TV/videos viewing) and all-cause mortality and CVD, but no evidence for mortality from cancer.”

**Level of Evidence for All-Cause Mortality and CVD mortality - Strong Evidence**

**Level of Evidence for Cancer Mortality – No evidence**

**Thorp AA, Owen N, Neuhaus M, Dunstan DW (2011) Sedentary behaviors and subsequent health outcomes in adults a systematic review of longitudinal studies, 1996-2011. Am J Prev Med 41: 207-215.**

**AMSTAR score - 4**

**Was scientific quality assessed and documented? Yes**

**Were conclusions formulated based on high methodological quality studies?** Yes

**Were the conclusions were based in longitudinal studies?** Yes

**Was physical activity extracted as covariate?** Yes - (34/48) = 71%

**Results/Conclusion**: “Three studies investigated the relationship between sitting time and mortality. With the exception of one study that reported associations for men only, time spent in sedentary behavior has been shown to be consistently associated with increased risk for all-cause, CVD-related, and all-other-causes mortality in both men and in women, independent of BMI and physical activity. Of the four studies that additionally examined cancer-related mortality risk, only one reported a signifıcant association with self-reported sitting time (in women only), which was independent of BMI and physical activity. Based on these fındings, there is a convincing level of evidence that a longitudinal relationship exists between sedentary behavior and all-cause, CVD-related, and all-other-causes mortality risk in men and women.”

**Level of Evidence for All-Cause Mortality, CVD mortality and All-other causes mortality - Strong Evidence**

**Wilmot EG, Edwardson CL, Achana FA, Davies MJ, Gorely T, et al. (2012) Sedentary time in adults and the association with diabetes, cardiovascular disease and death: systematic review and meta-analysis. Diabetologia 55: 2895-2905.**

**AMSTAR score - 9**

**Was scientific quality assessed and documented? Yes**

**Were conclusions formulated based on high methodological quality studies?** Yes

**Does the conclusions were based in longitudinal studies?** Yes

**Was physical activity extracted as covariate?** Yes - (10/18) = 56%

**Results/Conclusion**: “Of the remaining studies, 8 examined the association between sedentary time (TV viewing, sitting time, screen-time, TV and video) and all-cause mortality and 8 examined the association between sedentary time (TV and video, sitting time, TV and car sitting, TV and screen-time, TV viewing) and cardiovascular disease mortality. Greater sedentary time was associated with cardiovascular mortality and all-cause mortality.”

**Level of Evidence for All-Cause Mortality and CVD mortality - Strong Evidence**

**Ford ES, Caspersen CJ (2012) Sedentary behaviour and cardiovascular disease: a review of prospective studies. Int J Epidemiol 41: 1338-1353.**

**AMSTAR score – 4**

**Was scientific quality assessed and documented? No**

**Were conclusions formulated based on high methodological quality studies?** No

**Does the conclusions were based in longitudinal studies?** Yes

**Was physical activity extracted as covariate?** Yes - (11/11) = 100%

**Results/Conclusion**: “Our review of prospective studies found a significant association between screen time and cardiovascular mortality independent of levels of physical activity. Furthermore, two large cohort studies that lent themselves to a meta-analytic summary also suggested a significant increased risk between the time spent sitting and cardiovascular mortality, also independent of physical activity. Despite the need to solidify the evidence base concerning the health risks caused by excessive sedentary behaviour, available data are clear that being in the least active group is not desirable, especially when excessive sedentary behaviour is likely to comprise membership in that group”.

**Level of Evidence for All-Cause Mortality and CVD mortality - Strong Evidence**

**van Uffelen JGZ, Wong J, Chau JY, van der Ploeg HP, Riphagen I, et al. (2010) Occupational Sitting and Health Risks A Systematic Review. Am J Prev Med 39: 379-388.**

**AMSTAR score - 5**

**Was scientific quality assessed and documented? Yes**

**Were conclusions formulated based on high methodological quality studies?** Yes

**Does the conclusions were based in longitudinal studies?** Yes

**Was physical activity extracted as covariate?** Yes - (19/43) = 44%

**Results/Conclusion**: “Six prospective studies examined the association of occupational sitting with all-cause mortality, cardiovascular mortality and cancer mortality. In summary, four prospective studies found that sitting in the work was associated with an increased mortality risk (CVD mortality and all-cause mortality) , one study found no association (cancer, CVD and all-cause mortality), and one study found that sitting was associated with a decreased mortality risk.”

**Level of Evidence for All-Cause Mortality and CVD - Strong Evidence**

**CARDIOVASCULAR DISEASE – Sufficient Evidence**

**TV, screen-time and sitting time evidences were based on Wilmot et al.**

**Occupational sitting time was based on van Uffelen**

**Grontved A, Hu FB (2011) Television viewing and risk of type 2 diabetes, cardiovascular disease, and all-cause mortality: a meta-analysis. JAMA 305: 2448-2455.**

**AMSTAR score - 6**

**Was scientific quality assessed and documented? No**

**Were conclusions formulated based on high methodological quality studies?** No

**Were the conclusions were based in longitudinal studies?** Yes

**Was physical activity extracted as covariate?** Yes - (7/8) = 88%

**Results/Conclusion**: “Our results from the meta-analysis of prospective cohort studies suggest that TV viewing is consistently associated with higher risk of type 2 diabetes, fatal or nonfatal cardiovascular disease, and all-cause mortality.”

**Level of Evidence for CVD - Strong Evidence**

**Thorp AA, Owen N, Neuhaus M, Dunstan DW (2011) Sedentary behaviors and subsequent health outcomes in adults a systematic review of longitudinal studies, 1996-2011. Am J Prev Med 41: 207-215.**

**AMSTAR score - 4**

**Was scientific quality assessed and documented? Yes**

**Were conclusions formulated based on high methodological quality studies?** Yes

**Were the conclusions were based in longitudinal studies?** Yes

**Was physical activity extracted as covariate?** Yes - (34/48) = 71%

**Results/Conclusion**: “In general, consistent fındings exist with respect to engaging in high levels of sedentary behavior (unispecified) and increased risk for cardiovascular disease. However, caution is warranted in the interpretation of these fındings as the conclusions drawn are based on a limited number of studies. Further studies are required to confirm a longitudinal relationship.”

**Level of Evidence for CVD - Moderate Evidence**

**Wilmot EG, Edwardson CL, Achana FA, Davies MJ, Gorely T, et al. (2012) Sedentary time in adults and the association with diabetes, cardiovascular disease and death: systematic review and meta-analysis. Diabetologia 55: 2895-2905.**

**AMSTAR score - 9**

**Was scientific quality assessed and documented? Yes**

**Were conclusions formulated based on high methodological quality studies?** Yes

**Does the conclusions were based in longitudinal studies?** Yes

**Was physical activity extracted as covariate?** Yes - (10/18) = 56%

**Results/Conclusion**: “Of the remaining studies, three examined the association between sedentary time (TV viewing, sitting time, TV and screen-time) and cardiovascular disease. Greater sedentary time was associated with increasily risk for cardiovascular disease.”

**Level of Evidence for CVD - Strong Evidence**

**Ford ES, Caspersen CJ (2012) Sedentary behaviour and cardiovascular disease: a review of prospective studies. Int J Epidemiol 41: 1338-1353.**

**AMSTAR score – 4**

**Was scientific quality assessed and documented? No**

**Were conclusions formulated based on high methodological quality studies?** No

**Does the conclusions were based in longitudinal studies?** Yes

**Was physical activity extracted as covariate?** Yes - (11/11) = 100%

**Results/Conclusion**: “For six studies of screen time and CVD, the summary hazard ratio per 2-h increase was 1.17 (95% CI: 1.13–1.20). For two studies of sitting time, the summary hazard ratio per 2-h increase was 1.05 (95% CI: 1.01–1.09).” Our review of prospective studies found a significant association between screen time and cardiovascular mortality independent of levels of physical activity. Furthermore, two large cohort studies that lent them- selves to a meta-analytic summary also suggested a significant increased risk between the time spent sitting and cardiovascular mortality, also independent of physical activity. Despite the need to solidify the evidence base concerning the health risks caused by excessive sedentary behaviour, available data are clear that being in the least active group is not desirable, especially when excessive sedentary behaviour is likely to comprise membership in that group.”

**Level of Evidence for CVD - Strong Evidence**

**van Uffelen JGZ, Wong J, Chau JY, van der Ploeg HP, Riphagen I, et al. (2010) Occupational Sitting and Health Risks A Systematic Review. Am J Prev Med 39: 379-388.**

**AMSTAR score - 5**

**Was scientific quality assessed and documented? Yes**

**Were conclusions formulated based on high methodological quality studies?** Yes

**Does the conclusions were based in longitudinal studies?** Yes

**Was physical activity extracted as covariate?** Yes - (19/43) = 44%

**Results/Conclusion**: “All studies used self-report, categoric measure of occupational activity with sedentary, or mainly sitting, or physically very easy sittiing office work as one of the response options. In summary, the CVD papers showed conflicting results, with four showing an increased risk of CVD outcomes with occupational sitting, three showing no association, and one showing the opposite effect of increassed CVD risk with increasing occupational activity.”

**Level of Evidence for CVD - Insufficient Evidence**

**CANCER – Insufficient Evidence**

**Breast – Occupational sitting time was based in van Uffelen;**

**Colorectal - Occupational sitting time was based in van Uffelen; Unspecified based in Thorp**

**Cólon – Unspecified based in Thorp**

**Endometrial – Unspecified based on Proper;**

**Ovarian - Occupational sitting time was based in van Uffelen; Unspecified based in Thorp**

**Prostate – Conclusion based on Lynch et al.**

**Lynch BM (2010) Sedentary behavior and cancer: a systematic review of the literature and proposed biological mechanisms. Cancer Epidemiol Biomarkers Prev 19: 2691-2709.**

**AMSTAR score - 4**

**Was scientific quality assessed and documented? No**

**Were conclusions formulated based on high methodological quality studies?** No

**Were the conclusions were based in longitudinal studies?** Yes

**Was physical activity extracted as covariate?** Yes - (14/18) = 78%

**Results/Conclusion**: Sedentary behavior and cancer risk. Six of the 11 cancer risk studies were prospective cohort studies, four were case-control studies, and one was a randomized controlled trial. Insufficient evidence has accumulated to draw strong conclusions about associations between sedentary behavior and cancer.

**Level of Evidence for Cancer - Insufficient Evidence**

**Proper KI, Singh AS, van Mechelen W, Chinapaw MJM (2011) Sedentary behaviors and health outcomes among adults: A systematic review of prospective studies. American Journal of Preventive Medicine 40: 174-182.**

**AMSTAR score - 6**

**Was scientific quality assessed and documented? Yes**

**Were conclusions formulated based on high methodological quality studies?** Yes

**Were the conclusions were based in longitudinal studies?** Yes

**Was physical activity extracted as covariate?** Yes - (12/19) = 63%

**Results/Conclusion:** “Two high quality studies investigated the relationship between sedentary behavior (time spent sitting outside work; time spent sitting) and a specific type of cancer, namely, endometrial cancer. Based on the inconsistencies found between and within the two studies identified, there is insufficient evidence for the relationship between sedentary behavior and endometrial cancer.

**Level of Evidence for Cancer - Insufficient Evidence**

**Thorp AA, Owen N, Neuhaus M, Dunstan DW (2011) Sedentary behaviors and subsequent health outcomes in adults a systematic review of longitudinal studies, 1996-2011. Am J Prev Med 41: 207-215.**

**AMSTAR score - 4**

**Was scientific quality assessed and documented? Yes**

**Were conclusions formulated based on high methodological quality studies?** Yes

**Were the conclusions were based in longitudinal studies?** Yes

**Was physical activity extracted as covariate?** Yes - (34/48) = 71%

**Results/Conclusions:** “In general, consistent fındings exist with respect to engaging in high levels of sedentary behavior and increased risk for site-specifıc cancers; including ovarian, colon, and endometrial cancer. However, the extent of the mediating effect of both BMI and time spent in physical activity in these associations has not fully been elucidated. At present, there is limited evidence to conclude that a longitudinal relationship exists between sedentary behavior and cancer incidence.

**Level of Evidence for Ovarian, Colon and Endometrial Cancer - Moderate Evidence**

**Boyle T (2012) Physical Activity and Colon Cancer: Timing, Intensity, and Sedentary Behavior. Am J Lifestyle Med 6: 11.**

**AMSTAR score - 3**

**Was scientific quality assessed and documented? No**

**Were conclusions formulated based on high methodological quality studies?** No

**Were the conclusions were based in longitudinal studies?** Yes

**Was physical activity extracted as covariate?** No

**Results/Conclusions: “**The majority of studies that have investigated the effect of some kind of sedentary behavior (sitting) on colon cancer have shown an increased risk. However, much of this evidence comes from studies that have based occupational activity on job title, a method that may mistakenly classify jobs requiring a low level of activity but not prolonged sitting as sedentary.6 In addition, many of the studies that have investigated this issue have been hospital-based case–control studies, which are more prone to problems such as selection bias and recall bias and/or have been small studies with insufficient power to detect a statistically significant difference. Furthermore, few studies have assessed the potential confounding effect of aerobic physical activity, although 4 of the 6 studies that have adjusted for physical activity found that sedentary behavior was associated with an increased risk of colon cancer.”

**Level of Evidence for Cancer - Insufficient Evidence**

**van Uffelen JGZ, Wong J, Chau JY, van der Ploeg HP, Riphagen I, et al. (2010) Occupational Sitting and Health Risks A Systematic Review. Am J Prev Med 39: 379-388.**

**AMSTAR score - 5**

**Was scientific quality assessed and documented? Yes**

**Were conclusions formulated based on high methodological quality studies?** Yes

**Does the conclusions were based in longitudinal studies?** Yes

**Was physical activity extracted as covariate?** Yes - (19/43) = 44%

**Results/Conclusion:** “In summary, of the 17 studies, only five found that occupational sitting was associated with higher risk of breast cancer, ovarian cancer or colorectal cancer. Four of these studies were case-control studies, with on prospective study. Ten prospective studies found no evidence of an association, and two studies observed an increased lung cancer risk in people who were more active at work, compared with those in sedentary jobs.

**Level of Evidence for Breast, Ovarian, Colorectal Cancer - Insufficient Evidence**

**TYPE 2 DIABETES – Sufficient Evidence**

**Occupational sitting time was based in van Uffelen**

**TV and Screen-time time were based in Wilmot**

**Sitting was based in Proper**

**Grontved A, Hu FB (2011) Television viewing and risk of type 2 diabetes, cardiovascular disease, and all-cause mortality: a meta-analysis. JAMA 305: 2448-2455.**

**Was scientific quality assessed and documented? No**

**Were conclusions formulated based on high methodological quality studies?** No

**Were the conclusions were based in longitudinal studies?** Yes

**Was physical activity extracted as covariate?** Yes - (7/8) = 88%

**Results/Conclusion**: “Our results from the meta-analysis of prospective cohort studies suggest that TV viewing is consistently associated with higher risk of type 2 diabetes, fatal or nonfatal cardiovascular disease, and all-cause mortality.”

**Level of Evidence - Moderate Evidence**

**Proper KI, Singh AS, van Mechelen W, Chinapaw MJM (2011) Sedentary behaviors and health outcomes among adults: A systematic review of prospective studies. American Journal of Preventive Medicine 40: 174-182.**

**AMSTAR score - 6**

**Was scientific quality assessed and documented? Yes**

**Were conclusions formulated based on high methodological quality studies?** Yes

**Were the conclusions were based in longitudinal studies?** Yes

**Was physical activity extracted as covariate?** Yes - (12/19) = 63%

**Results/Conclusion**: “Two low-quality prospective studies investigated the relationship between sedentary behavior and incidence of type 2 diabetes in men and women, respectively. The study of Hu et al. reported a significant relationship between the time spent TV viewing and type 2 diabetes among 37,918 male health professionals. In 2003, Hu et al., reported the relationship over a 6-year period between various sedentary behaviors and incidence of diabetes type 2 in 68,497 women. Theu found significant positive relationship for TV viewing, sitting at work or away from home or driving, and other sitting at homw with those viewing TV for at least 6 hours per week being at increased risk of developing diabetes type 2 compared to those viewing 0-1 hour per week. Based on te consistent findings of these two low-quality studies, there is moderate evidence for a significant positive relationship between the time spent sitting and the risk for type 2 diabetes.”

**Level of Evidence - Moderate Evidence**

**Thorp AA, Owen N, Neuhaus M, Dunstan DW (2011) Sedentary behaviors and subsequent health outcomes in adults a systematic review of longitudinal studies, 1996-2011. Am J Prev Med 41: 207-215.**

**Was scientific quality assessed and documented? Yes**

**Were conclusions formulated based on high methodological quality studies?** Yes

**Were the conclusions were based in longitudinal studies?** Yes

**Was physical activity extracted as covariate?** Yes - (34/48) = 71%

**Results/Conclusion:** “In general, consistent findings exist with respect to enganing in high levels of sedentary behavior and increased risk for diabetes. However, the extent of the mediating effect of both BMI and time spent in physical activity in these associations has not fully been elucidated. At present, there is limited evidence to conclude that a longitudinal relationship exists between sedentary behavior and risk for type 2 diabetes.”

**Level of Evidence - Insufficient Evidence**

**Wilmot EG, Edwardson CL, Achana FA, Davies MJ, Gorely T, et al. (2012) Sedentary time in adults and the association with diabetes, cardiovascular disease and death: systematic review and meta-analysis. Diabetologia 55: 2895-2905.**

**AMSTAR score - 9**

**Was scientific quality assessed and documented? Yes**

**Were conclusions formulated based on high methodological quality studies?** Yes

**Does the conclusions were based in longitudinal studies?** Yes

**Was physical activity extracted as covariate?** Yes - (10/18) = 56%

**Results/Conclusion**: “Of the remaining studies, ten examined the association between sedentary time (TV viewing, TV and screen-time, TV and video) and type 2 diabetes. Greater sedentary time was associated with increasily risk for type 2 diabetes.”

**Level of Evidence - Strong Evidence**

**van Uffelen JGZ, Wong J, Chau JY, van der Ploeg HP, Riphagen I, et al. (2010) Occupational Sitting and Health Risks A Systematic Review. Am J Prev Med 39: 379-388.**

**AMSTAR score - 5**

**Was scientific quality assessed and documented? Yes**

**Were conclusions formulated based on high methodological quality studies?** Yes

**Does the conclusions were based in longitudinal studies?** Yes

**Was physical activity extracted as covariate?** Yes - (19/43) = 44%

**Results/Conclusion:** “Four studies examined the association between occupational sitting and DM, of which one was a cross-sectional stidy and three were prospective studies. All studies used self-reported measures; three used categoric variable for occupational activity, with sedentary or physically very easy sitting office work as a response option and one used a continous measure of sitting time that was categorized for the analyses. Two studies used self-reported DM as the outcome, whereas the remainder derived data on DM from national registers or used DM as diagnosed by a dorctor or blood sample. In summary, for DM, two prospective studies and one cross-sectional study found that sitting was associated with increased risk of DM, whereas one prospective study found no association.

**Level of Evidence - Insufficient Evidence**

**METABOLIC SYNDROME AND INDIVIDUAL CARDIOVASCULAR RISK FACTORS -**

***METABOLIC SYNDROME* –**

**CHILDREN ADOLESCENTS – based on Tremblay**

**ADULTS –Based on Edwardson;**

**Salmon J, Tremblay MS, Marshall SJ, Hume C (2011) Health risks, correlates, and interventions to reduce sedentary behavior in young people. Am J Prev Med 41: 197-206.**

**AMSTAR Score – 3**

**Was scientific quality assessed and documented? No**

**Were conclusions formulated based on high methodological quality studies? No**

**Does the conclusions were based in longitudinal studies?** No

**Was physical activity extracted as covariate?** No

**Results/Conclusion:** More daily screentime were independetly associated with an increased likehood of Canadian adolescents having metabolic syndrome.

**Level of Evidence - Moderate Evidence (Adolescents)**

**Tremblay MS, LeBlanc AG, Kho ME, Saunders TJ, Larouche R, et al. (2011) Systematic review of sedentary behaviour and health indicators in school-aged children and youth. Int J Behav Nutr Phys Act 8:98. doi: 10.1186/1479-5868-8-98.**

**AMSTAR Score - 9**

**Was scientific quality assessed and documented? Yes**

**Were conclusions formulated based on high methodological quality studies? Yes**

**Does the conclusions were based in longitudinal studies?** Yes

**Was physical activity extracted as covariate?** No

**Results/Conclusion:** Eleven studies assessed the relationship between time spent engaging in sedentary behaviour and risk factors for MS and CVD. All of the studies reported that increased sedentary time was associated with increased risk for MS or CVD. However, the results of these studies should be viewed with caution as the proportion of children and youth who have measurable health risk factors for MS or CVD is quite low. Longitudinal studies found that those watching more than 2 hours of television per day had higher serum cholesterol levels and were more likely to have high blood pressure than their peers who watched less TV. Cross sectional studies reported that high levels of screen time and self-reported sedentary behaviour were associated with increased risk for high systolic and diastolic blood pressure, higher HbA1 c, fasting insulin, insulin resistance, and MS. These risk factors increase in a dose response manner with increased screen time. One cross sectional study reported a significant relationship between watching TV and increased cholesterol in adolescents, but not in younger children. The level of evidence for MS and CVD risk factors was classified as Level 3 with a mean Downs and Black score of 21.7 (standard deviation: ± 2.1), indicating moderate quality of reporting. In summary, increased screen time is associated with increased risk for markers of metabolic syndrome and cardiovascular disease. Risk increases in a dose-response manner.

**ADULTS**

**Edwardson CL, Gorely T, Davies MJ, Gray LJ, Khunti K, et al. (2012) Association of sedentary behaviour with metabolic syndrome: a meta-analysis. PLoS One 7: e34916.**

**AMSTAR Score - 9**

**Was scientific quality assessed and documented? Yes**

**Were conclusions formulated based on high methodological quality studies? Yes**

**Does the conclusions were based in longitudinal studies?** Yes

**Was physical activity extracted as covariate?** Yes – (8/10) = 80%

**Results/Conclusion:** Greater time spent sedentary increased the odds of metabolic syndrome. The results remained largely unchanged after conducting a sensitivity analysis of those studies which adjusted for physical activity. There were no differences for subgroups of sex, sedentary measure, metabolic syndrome definition, study quality or country income.

**Level of Evidence - Strong Evidence**

***INDIVIDUAL CARDIOVASCULAR RISCK FACTORS***

***CHILDREN – BASED ON TRAMBLEY ET AL.***

***ADULTS – BASED ON PROPER ET AL. AND***

**Chinapaw MJ, Proper KI, Brug J, van Mechelen W, Singh AS (2011) Relationship between young peoples' sedentary behaviour and biomedical health indicators: a systematic review of prospective studies. Obes Rev 12: e621-632.**

**AMSTAR Score - 7**

**Was scientific quality assessed and documented? Yes**

**Were conclusions formulated based on high methodological quality studies? Yes**

**Does the conclusions were based in longitudinal studies?** Yes

**Was physical activity extracted as covariate?** Yes - (14/31) = 45%

**Results/Conclusion:** Few studies examined the longitudinal relationship between sedentary time and other health outcomes such as blood pressure, blood lipids, ﬁtness/VO2max or bone mass. Based on these ﬁndings, we can conclude that there is moderate evidence for a longitudinal inverse relationship between sedentary time and aerobic ﬁtness (based on one high-quality and two low- quality studies) and insufﬁcient evidence for a longitudinal relationship between sedentary time and blood pressure, blood lipids or bone mass.

**Level of Evidence - Moderate Evidence**

**Tremblay MS, LeBlanc AG, Kho ME, Saunders TJ, Larouche R, et al. (2011) Systematic review of sedentary behaviour and health indicators in school-aged children and youth. Int J Behav Nutr Phys Act 8:98. doi: 10.1186/1479-5868-8-98.**

**AMSTAR Score - 10**

**Was scientific quality assessed and documented? Yes**

**Were conclusions formulated based on high methodological quality studies? Yes**

**Does the conclusions were based in longitudinal studies?** Yes

**Was physical activity extracted as covariate?** No

**Results/Conclusion:** Eleven studies assessed the relationship between time spent engaging in sedentary behaviour and risk factors for MS and CVD. All of the studies reported that increased sedentary time was associated with increased risk for MS or CVD. However, the results of these studies should be viewed with caution as the proportion of children and youth who have measurable health risk factors for MS or CVD is quite low. Longitudinal studies found that those watching more than 2 hours of television per day had higher serum cholesterol levels and were more likely to have high blood pressure than their peers who watched less TV. Cross sectional studies reported that high levels of screen time and self-reported sedentary behaviour were associated with increased risk for high systolic and diastolic blood pressure, higher HbA1 c, fasting insulin, insulin resistance, and MS. These risk factors increase in a dose response manner with increased screen time. One cross sectional study reported a significant relationship between watching TV and increased cholesterol in adolescents, but not in younger children. The level of evidence for MS and CVD risk factors was classified as Level 3 with a mean Downs and Black score of 21.7 (standard deviation: ± 2.1), indicating moderate quality of reporting. In summary, increased screen time is associated with increased risk for markers of metabolic syndrome and cardiovascular disease. Risk increases in a dose-response manner.

**Level of Evidence - Insufficient Evidence**

**Proper KI, Singh AS, van Mechelen W, Chinapaw MJM (2011) Sedentary behaviors and health outcomes among adults: A systematic review of prospective studies. American Journal of Preventive Medicine 40: 174-182.**

**AMSTAR score - 6**

**Was scientific quality assessed and documented? Yes**

**Were conclusions formulated based on high methodological quality studies?** Yes

**Were the conclusions were based in longitudinal studies?** Yes

**Was physical activity extracted as covariate?** Yes - (12/19) = 63%

**Results/Conclusion**: Four high-quality prospective studies examied the relationship between sedentary behavior (TV viewing, PC use, driving, objectively mesured sedentary time) and CVD risk factors. Based on the finddings of the studies identified, there is insufficient evidence for a significant relationship between sedentary behavior and various CVD risk factors.

**Level of Evidence - Insufficient Evidence**

**Thorp AA, Owen N, Neuhaus M, Dunstan DW (2011) Sedentary behaviors and subsequent health outcomes in adults a systematic review of longitudinal studies, 1996-2011. Am J Prev Med 41: 207-215.**

**AMSTAR score - 4**

**Was scientific quality assessed and documented? Yes**

**Were conclusions formulated based on high methodological quality studies?** Yes

**Were the conclusions were based in longitudinal studies?** Yes

**Was physical activity extracted as covariate?** Yes - (34/48) = 71%

**Results/Conclusion:** There is insufficient evidence to conclude that a longitudinal relationship exists between sedentary behavior, markers of cardiometabolic health, and metabolic conditions.

**Level of Evidence - Insufficient Evidence**

**OBESITY, OVERWEIGHT AND ADIPOSITY**

***CHILDREN AND ADOLESCENTS – Based on Tremblay***

**Tremblay MS, LeBlanc AG, Kho ME, Saunders TJ, Larouche R, et al. (2011) Systematic review of sedentary behaviour and health indicators in school-aged children and youth. Int J Behav Nutr Phys Act 8:98. doi: 10.1186/1479-5868-8-98.**

**AMSTAR Score - 10**

**Was scientific quality assessed and documented? Yes**

**Were conclusions formulated based on high methodological quality studies? Yes**

**Does the conclusions were based in longitudinal studies?** Yes

**Was physical activity extracted as covariate?** No

**Results/Conclusion:** The level of evidence reporting on the relationship between sedentary behaviour and body composition was of moderate quality and level of evidence 2 (Randomized control trials with important limitations).

**Level of Evidence – Strong Evidence**

**Chinapaw MJ, Proper KI, Brug J, van Mechelen W, Singh AS (2011) Relationship between young peoples' sedentary behaviour and biomedical health indicators: a systematic review of prospective studies. Obes Rev 12: e621-632.**

**AMSTAR Score - 7**

**Was scientific quality assessed and documented? Yes**

**Were conclusions formulated based on high methodological quality studies? Yes**

**Does the conclusions were based in longitudinal studies?** Yes

**Was physical activity extracted as covariate?** Yes - (14/31) = 45%

**Results/Conclusion: “**Twenty-six studies examined the longitudinal relationship between sedentary time and BMI or BMI z-score. All studies looked at TV viewing and included computer use as well. Four of six high-quality studies observed a signiﬁcant positive relationship between sedentary time and BMI of which one study in boys only. Two high- quality studies in girls only found no signiﬁcant association between sedentary time and BMI. According to the best evidence synthesis we found insufﬁcient evidence for a positive longitudinal relationship between self- or proxy- reported sedentary time and BMI when considering boys and girls together. However, for girls, it appeared that there was no evidence for such a relationship. Ten studies examined sedentary time in relation to indicators of fat mass such as waist circumference, fat percentage or skinfold thickness. Five studies found a signiﬁcant positive relationship while ﬁve did not. Two high-quality studies found a signiﬁcant positive relationship, one in girls but not boys and one in boys but not girls. Two other high-quality studies in girls only found no signiﬁcant relationship meaning that we found insufﬁcient evidence for a longitudinal relationship between self- or proxy-reported sedentary time and indicators of fat mass.

**Level of Evidence – Insufficient Evidence**

**Costigan SA, Barnett L, Plotnikoff RC, Lubans DR (2013) The health indicators associated with screen-based sedentary behavior among adolescent girls: a systematic review. J Adolesc Health 52: 382-392.**

**AMSTAR Score - 6**

**Was scientific quality assessed and documented? Yes**

**Were conclusions formulated based on high methodological quality studies? Yes**

**Does the conclusions were based in longitudinal studies?** Yes

**Was physical activity extracted as covariate?** Yes - (16/33) = 49%

**Results/Conclusion:** Most of the studies (19/33, 58%) examined the association between screen-based sedentary behavior and weight status. Indicators of screen-based sedentary behavior included increased body mass index (BMI)/body fatness, increased risk of overweight/obesity, and increased odds of obesity. These have all been grouped as weight status. Only one of these studies reported no association between screen-based sedentary behavior and weight status. When additional summary coding was completed for low risk of bias studies only (e.g., eight studies gained a risk of bias score >4) a strong association was found for weight status (i.e., 88% of low risk of bias studies [n = 7/8] reported a positive association between screen-based sedentary behavior and increasing weight status.

**Level of Evidence – Strong Evidence**

**LeBlanc AG, Spence JC, Carson V, Connor Gorber S, Dillman C, et al. (2012) Systematic review of sedentary behaviour and health indicators in the early years (aged 0-4 years). Appl Physiol Nutr Metab 37: 753-772.**

**AMSTAR Score - 8**

**Was scientific quality assessed and documented? Yes**

**Were conclusions formulated based on high methodological quality studies? Yes**

**Does the conclusions were based in longitudinal studies?** Yes

**Was physical activity extracted as covariate?** No

**Results/Conclusion:** “In conclusion, this review found low- to moderate-quality evidence to suggest that increased television viewing is associated with unfavourable measures of adiposity”

**Level of Evidence – Moderate Evidence**

**Marshall SJ, Biddle SJH, Gorely T, Cameron N, Murdey I (2004) Relationships between media use, body fatness and physical activity in children and youth: a meta-analysis. Int J Obes 28: 1238-1246.**

**AMSTAR Score - 3**

**Was scientific quality assessed and documented? No**

**Were conclusions formulated based on high methodological quality studies? No**

**Does the conclusions were based in longitudinal studies?** Yes

**Was physical activity extracted as covariate?** No

**Results/Conclusion:** It is concluded that a statistically small relationship exists between TV viewing and body fatness among children and youth although the magnitude of the relationship suggests that we should be cautious about the clinical relevance of this finding

**Level of Evidence – Moderate Evidence**

**Prentice-Dunn H, Prentice-Dunn S (2012) Physical activity, sedentary behavior, and childhood obesity: A review of cross-sectional studies. Psy Health Med 17: 255-273.**

**AMSTAR Score - 2**

**Was scientific quality assessed and documented? No**

**Were conclusions formulated based on high methodological quality studies? No**

**Does the conclusions were based in longitudinal studies?** No

**Was physical activity extracted as covariate?** No

**Results/Conclusion:** In general, sedentary behaviors were positively associated with weight status. However, gender diﬀerences appeared in some studies for each variable.

**Level of Evidence – Strong Evidence**

**Rey-Lopez JP, Vicente-Rodriguez G, Biosca M, Moreno LA (2008) Sedentary behaviour and obesity development in children and adolescents. Nutr Metab Cardiovasc Dis 18: 242-251.**

**AMSTAR Score – 3**

**Was scientific quality assessed and documented? No**

**Were conclusions formulated based on high methodological quality studies? No**

**Does the conclusions were based in longitudinal studies?** Yes

**Was physical activity extracted as covariate?** No

**Results/Conclusion:** The *causes* of excessive weight gain in children are multi-factorial [13]. With regard to environmental factors, sufficient evidence exists to recommend setting a limit to the time spent watching TV, especially by young children.

**Level of Evidence – Strong Evidence**

**Rossi C, Albernaz D, Vasconcelos F, Assis M, Di Pietro P (2010) Television influence on food intake in children and adolescents: a systematic review. Rev Nutr 23: 13.**

**AMSTAR - 3**

**Was scientific quality assessed and documented? No**

**Were conclusions formulated based on high methodological quality studies? No**

**Does the conclusions were based in longitudinal studies?** No

**Was physical activity extracted as covariate?** No

**Results/Conclusion:** Since time spent watching television is associated with unhealthy food habits and reduced levels of physical activity, it becomes an important factor in the promotion of obesity in children and adolescents.

**Level of Evidence – Strong Evidence**

**Salmon J, Tremblay MS, Marshall SJ, Hume C (2011) Health risks, correlates, and interventions to reduce sedentary behavior in young people. Am J Prev Med 41: 197-206.**

**AMSTAR Score - 3**

**Was scientific quality assessed and documented? No**

**Were conclusions formulated based on high methodological quality studies? No**

**Does the conclusions were based in longitudinal studies?** No

**Was physical activity extracted as covariate?** No

**Results/Conclusion:** Reviews of evidence from cross-setional, longitudinal, and intervention studies have shown primarily consistent associations between screen time and overweight/obesity in children and youth.

**Level of Evidence – Strong Evidence**

**Velde SJT, van Nassau F, Uijtdewilligen L, van Stralen MM, Cardon G, et al. (2012) Energy balance-related behaviours associated with overweight and obesity in preschool children: a systematic review of prospective studies. Obes Rev 13: 56-74.**

**AMSTAR Score - 7**

**Was scientific quality assessed and documented? Yes**

**Were conclusions formulated based on high methodological quality studies? Yes**

**Does the conclusions were based in longitudinal studies?** Yes

**Was physical activity extracted as covariate?** No

**Results/Conclusion:** “Moderate evidence was observed for a positive association between television viewing and overweight.”

**Level of Evidence – Moderate Evidence**

***ADULTS – Based on Proper (TV) and van Uffelen (occupational sitting)***

**Proper KI, Singh AS, van Mechelen W, Chinapaw MJM (2011) Sedentary behaviors and health outcomes among adults: A systematic review of prospective studies. American Journal of Preventive Medicine 40: 174-182.**

**AMSTAR score - 6**

**Was scientific quality assessed and documented? Yes**

**Were conclusions formulated based on high methodological quality studies?** Yes

**Were the conclusions were based in longitudinal studies?** Yes

**Was physical activity extracted as covariate?** Yes - (12/19) = 63%

**Results/Conclusion**: “Four prospective studies were identified that examined the relationship between sedentary behavior (sedentary work, TV viewing) and the risk for overweight or obesity. Based on the incosistent findings among the studies, there is insufficient evidence for the relationship between sedentary behavior and the risk for overweight or obesity. Only one low-quality prospective study examined the relationship between sedentary behavior and waist gain, both measured by self-report. Based on this single study, there is insufficient evidence for the relationship between sedentary behavior and waist gains.”

**Level of Evidence – Insufficient Evidence**

**Thorp AA, Owen N, Neuhaus M, Dunstan DW (2011) Sedentary behaviors and subsequent health outcomes in adults a systematic review of longitudinal studies, 1996-2011. Am J Prev Med 41: 207-215.**

**Was scientific quality assessed and documented? Yes**

**Were conclusions formulated based on high methodological quality studies?** Yes

**Were the conclusions were based in longitudinal studies?** Yes

**Was physical activity extracted as covariate?** Yes - (34/48) = 71%

**Results/Conclusion:** “Based on the findings, there is limited evidence that a longitudinal relationship exist between sedentary behavior, weight gain, and risk of obesity in adults.

**Level of Evidence – Insufficient Evidence**

**van Uffelen JGZ, Wong J, Chau JY, van der Ploeg HP, Riphagen I, et al. (2010) Occupational Sitting and Health Risks A Systematic Review. Am J Prev Med 39: 379-388.**

**AMSTAR score - 5**

**Was scientific quality assessed and documented? Yes**

**Were conclusions formulated based on high methodological quality studies?** Yes

**Does the conclusions were based in longitudinal studies?** Yes

**Was physical activity extracted as covariate?** Yes - (19/43) = 44%

**Results/Conclusion: “**In summary, five of ten cross-sectional studies showed a positive association between occupational sitting and BMI, but four studies found no association and one study found a negative association. Of the three prospective studies, one found a positive association, bet the other two found no association.”

**Level of Evidence – Insufficient Evidence**

**MENTAL HEALTH AND COGNITIVE ASPECTS**

***MENTAL DISORDERS IN CHILDREN – Based on LeBlanc***

**Costigan SA, Barnett L, Plotnikoff RC, Lubans DR (2013) The health indicators associated with screen-based sedentary behavior among adolescent girls: a systematic review. J Adolesc Health 52: 382-392.**

**AMSTAR Score - 6**

**Was scientific quality assessed and documented? Yes**

**Were conclusions formulated based on high methodological quality studies? Yes**

**Does the conclusions were based in longitudinal studies?** Yes

**Was physical activity extracted as covariate?** Yes - (16/33) = 49%

**Results/Conclusion:** “Six studies examined the relationship between screen-based sedentary behavior and psychosocial health (Table 3). A positive association between screen-based sedentary behavior and depressive symptoms was reported in three studies [51,54,72]. Four studies reported a negative association between screen- time activities and perceived health [10,55,72,73], and one study reported a negative association between screen time and psychological wellbeing. Screen-based sedentary behavior is associated with a range of adverse health consequences, but additional longitudinal studies are needed to better understand the health impacts”.

**Level of Evidence – Insufficient Evidence**

**LeBlanc AG, Spence JC, Carson V, Connor Gorber S, Dillman C, et al. (2012) Systematic review of sedentary behaviour and health indicators in the early years (aged 0-4 years). Appl Physiol Nutr Metab 37: 753-772.**

**AMSTAR Score - 8**

**Was scientific quality assessed and documented? Yes**

**Were conclusions formulated based on high methodological quality studies? Yes**

**Does the conclusions were based in longitudinal studies?** Yes

**Was physical activity extracted as covariate?** No

**Results/Conclusion:** “Five prospective cohort studies (Christakis and Zimmer- man 2007; Foster and Watkins 2010; Mistry et al. 2007; Pa- gani et al. 2010; Zimmerman et al. 2005) examined the relationship between television viewing and cognitive devel- opment in toddlers. Of these, 2 studies (Foster and Watkins 2010; Schmidt et al. 2009) reported no significant relation- ship. The remaining 3 studies (Christakis et al. 2009; Mistry et al. 2007; Pagani et al. 2010) reported a dose–response re- lationship with each additional hour of television exposure related to decreased vocalization, classroom engagement, and math scores. One low-quality prospective cohort study (Zim- merman and Christakis 2007) reported on the relationship be- tween television viewing and cognitive development in preschoolers. The studies examining cognitive outcomes for toddlers and preschoolers were low quality (Tables 3 and 4).”

**Level of Evidence – Moderate Evidence**

**Salmon J, Tremblay MS, Marshall SJ, Hume C (2011) Health risks, correlates, and interventions to reduce sedentary behavior in young people. Am J Prev Med 41: 197-206.**

**AMSTAR Score - 3**

**Was scientific quality assessed and documented? No**

**Were conclusions formulated based on high methodological quality studies? No**

**Does the conclusions were based in longitudinal studies?** No

**Was physical activity extracted as covariate?** No

**Results/Conclusion:** In summary, there is evidence sugesting that time spent sedentary, overal (as measure objectively) and in from of eletronic screens, is negatively associated with socognitive outcomes. Longitudinal and experimental evidence of associations between objectively assessed sedentary time and childrens health outcomes is needed.

**Level of Evidence – Insufficient Evidence**

***MENTAL AND DEPRESSIVE DISORDERS IN ADULTS –***

***Depressive Symptoms - Based on Thorp***

**Postnatal Depression – based on Teychenne**

**Teychenne M, Ball K, Salmon J (2010) Sedentary behavior and depression among adults: A review. Int J Behav Med 17: 246-254.**

**AMSTAR Score - 6**

**Was scientific quality assessed and documented? Yes**

**Were conclusions formulated based on high methodological quality studies? Yes**

**Were the conclusions were based in longitudinal studies?** Yes

**Was physical activity extracted as covariate?** No

**Results/Conclusion:** “When evaluated against published guidelines, it may be concluded that the available evidence for the effects of sedentary behavior on risk of depression in adults is limited by methodological weaknesses. While the findings of this review are not unequivocal, on the whole they are suggestive of a positive association between sedentary behaviors and risk of depression.”

**Level of Evidence – Insufficient Evidence**

**Thorp AA, Owen N, Neuhaus M, Dunstan DW (2011) Sedentary behaviors and subsequent health outcomes in adults a systematic review of longitudinal studies, 1996-2011. Am J Prev Med 41: 207-215.**

**AMSTAR score - 4**

**Was scientific quality assessed and documented? Yes**

**Were conclusions formulated based on high methodological quality studies?** Yes

**Were the conclusions were based in longitudinal studies?** Yes

**Was physical activity extracted as covariate?** Yes - (34/48) = 71%

**Results/Conclusion: “**Increased risk of cardiovascular disease, symptomatic gallstone disease, mental disorders, and hypertension were shown to be associated with time spent in sedentary behavior independently of physical activity time.”

**Level of Evidence – Insufficient Evidence**

**Teychenne M, York R (2013) Physical activity, sedentary behavior, and postnatal depressive symptoms: a review. Am J Prev Med 45: 217-227.**

**AMSTAR Score - 7**

**Was scientific quality assessed and documented? Yes**

**Were conclusions formulated based on high methodological quality studies? Yes**

**Were the conclusions were based in longitudinal studies?** No

**Was physical activity extracted as covariate?** No

**Results/Conclusion:** “Of the two observational studies that examined the relationship between sedentary behavior and the pres- ence of postnatal depressive symptoms, one found a positive association. That is, the greater the time spent engaged in sedentary behavior, the greater the presence of depressive symptoms. Although that cross-sectional study used a measure of sedentary behavior that encom- passed all domains (leisure, transport, work, personal care) and included activities such as light ofﬁce work (sitting); talking on the phone; reading; riding in a car or bus; reclining with baby, a number of the activities included in the analyses were and/or may have been nonsedentary (e.g., standing at work, listening to music). Further, no details were provided as to the dose of sedentary behavior that was linked to an increased presence of postnatal depressive symptoms.”

**Level of Evidence – Insufficient Evidence**

**MUSCULOSKELETAL DISORDERS**

***ADOLESCENTS – Based on Costigan***

**Costigan SA, Barnett L, Plotnikoff RC, Lubans DR (2013) The health indicators associated with screen-based sedentary behavior among adolescent girls: a systematic review. J Adolesc Health 52: 382-392.**

**AMSTAR Score - 6**

**Was scientific quality assessed and documented? Yes**

**Were conclusions formulated based on high methodological quality studies? Yes**

**Does the conclusions were based in longitudinal studies?** No

**Was physical activity extracted as covariate?** Yes - (16/33) = 49%

**Results/Conclusion:** The association between screen-based sedentary behavior and musculoskeletal pain was examined in two studies. One study found combined sources of screen time to be associated with increased neck, shoulder, and lower back pain, whereas an additional study reported television and computer use to be associated with increased backache and headache. The association between screen-based sedentary behavior and musculoskeletal pain was therefore rated as positive (i.e., all studies reported increased musculoskeletal pain).

**Level of Evidence – Insufficient Evidence**

**Chen SM, Liu MF, Cook J, Bass S, Lo SK (2009) Sedentary lifestyle as a risk factor for low back pain: a systematic review. Int Arch Occup Environ Health 82: 797-806.**

**AMSTAR Score - 5**

**Was scientific quality assessed and documented? Yes**

**Were conclusions formulated based on high methodological quality studies? Yes**

**Does the conclusions were based in longitudinal studies?**

**Was physical activity extracted as covariate?** No

**Results/Conclusion:** Only one high-quality cohort study reported a positive association between sitting at work and low back pain among schoolchildren. One nonhigh-quality study on schoolchildren reporeted a trend towards time spent playing computer games and low back pain. Therefore, little evidence exists that sedentary behavior or prolonged sitting at work and during leisure time is related to low back pain.

**Level of Evidence – Moderate Evidence**

***ADULTS –***

***Occupational computer Based on* Waersted et al.**

***Screen-time Based on* Chen et al.**

**Chen SM, Liu MF, Cook J, Bass S, Lo SK (2009) Sedentary lifestyle as a risk factor for low back pain: a systematic review. Int Arch Occup Environ Health 82: 797-806.**

**AMSTAR Score - 5**

**Was scientific quality assessed and documented? Yes**

**Were conclusions formulated based on high methodological quality studies? Yes**

**Does the conclusions were based in longitudinal studies?** Yes

**Was physical activity extracted as covariate?** No

**Results/Conclusion:** Therefore, little evidence exists that sedentary behavior or prolonged sitting at work and during leisure time is related to low back pain.

**Level of Evidence – Insufficient Evidence**

**IJmker S, Huysmans MA, Blatter BM, van der Beek AJ, van Mechelen W, et al. (2007) Should office workers spend fewer hours at their computer? A systematic review of the literature. Occup Environ Med 64: 211-222.**

**AMSTAR Score - 7**

**Was scientific quality assessed and documented? Yes**

**Were conclusions formulated based on high methodological quality studies? Yes**

**Does the conclusions were based in longitudinal studies?** Yes

**Was physical activity extracted as covariate?** No

**Results/Conclusion:** “This review showed moderate evidence for an association between the duration of mouse use and the incidence of hand– arm symptoms. Indications for a dose–response were found. In addition, the neck–shoulder region seemed less susceptible to exposure to computer use than the hand–arm region. Both findings are supported by a pathophysiological mechanism based on the overuse of muscles during computer use. The low number of high-quality studies prevents drawing a firm conclusion. More research is needed to confirm our findings.”

**Level of Evidence – Insufficient Evidence**

**Waersted M, Hanvold TN, Veiersted KB (2010) Computer work and musculoskeletal disorders of the neck and upper extremity: a systematic review. BMC Musculoskelet Disord 11: 79.**

**Was scientific quality assessed and documented? Yes**

**Were conclusions formulated based on high methodological quality studies? Yes**

**Does the conclusions were based in longitudinal studies?** Yes

**Was physical activity extracted as covariate? Yes - (4/22) = 15%**

**Results/Conclusion: “**There is limited epidemiological evidence for an association between aspects of computer work and some of the clinical diagnoses studies. Considered as moderate or strong and there is a need for more and better documentation.”

**Level of Evidence – Insufficient Evidence**

**OTHER BEHAVIOURS**

***CHILDREN***

***PHYSICAL ACTIVITY – Based on Marshall et al.***

**Marshall SJ, Biddle SJH, Gorely T, Cameron N, Murdey I (2004) Relationships between media use, body fatness and physical activity in children and youth: a meta-analysis. Int J Obes 28: 1238-1246.**

**AMSTAR Score - 3**

**Was scientific quality assessed and documented? No**

**Were conclusions formulated based on high methodological quality studies? No**

**Does the conclusions were based in longitudinal studies?** Yes

**Was physical activity extracted as covariate?** No

**Results/Conclusion:** The relationship between TV viewing and physical activity is small but negative. The sample-weighted fully corrected effect size was 0.141. This suggests that the relationship is best described as ‘small.’ Again, this should be interpreted with caution because the mean effect size is based on only 10 primary effects and second-order sampling error may be present.

**Level of Evidence – Insufficient Evidence**

**Rossi C, Albernaz D, Vasconcelos F, Assis M, Di Pietro P (2010) Television influence on food intake in children and adolescents: a systematic review. Rev Nutr 23: 13.**

**AMSTAR Score - 3**

**Was scientific quality assessed and documented? No**

**Were conclusions formulated based on high methodological quality studies? No**

**Does the conclusions were based in longitudinal studies?** No

**Was physical activity extracted as covariate?** No

**Results/Conclusion:** “In six of ten international studies, there was a positive association between watching television and obesity and a negative association between times spent watching television and doing physical activity.”

**Level of Evidence – Insufficient Evidence**

***AGGRESSION – Based on Tremblay et al.***

**Mitrofan O, Paul M, Spencer N (2009) Is aggression in children with behavioural and emotional difficulties associated with television viewing and video game playing? A systematic review. Child Care Health Dev 35: 5-15.**

**Was scientific quality assessed and documented? Yes**

**Were conclusions formulated based on high methodological quality studies? Yes**

**Does the conclusions were based in longitudinal studies?**

**Was physical activity extracted as covariate?** No

**Results/Conclusion:** “Overall, there are only a few, methodologically ﬂawed studies in this area. There is therefore a need for rigorous qualitative and quantitative research, especially adequately powered studies, and the development of valid and reliable measures. The need for accuracy and completeness in the reporting of studies is also key. We recognize, however, that the complexity of investigating aggression and amount and content of media consumption seems almost limitless

**Level of Evidence – No Evidence**

***UNHEALTHY DIETARY INTAKE – Based on Pearson et al.***

**Pearson N, Biddle SJH (2011) Sedentary behavior and dietary intake in children, adolescents, and adults: A systematic review. Am J Prev Med 41: 178-188.**

**AMSTAR Score - 5**

**Was scientific quality assessed and documented? Yes**

**Were conclusions formulated based on high methodological quality studies? Yes**

**Does the conclusions were based in longitudinal studies?**

**Was physical activity extracted as covariate?** No

**Results/Conclusion:** “The association drawn mainly from cross-sectional studies is that sedentary behavior, usually assessed as screen time and predominantly TV viewing, is associated with unhealthy dietary behaviors in children, adolescents and adults.”

**Level of Evidence – Insufficient Evidence**

**Rossi C, Albernaz D, Vasconcelos F, Assis M, Di Pietro P (2010) Television influence on food intake in children and adolescents: a systematic review. Rev Nutr 23: 13.**

**AMSTAR Score - 3**

**Was scientific quality assessed and documented? No**

**Were conclusions formulated based on high methodological quality studies? No**

**Does the conclusions were based in longitudinal studies?** No

**Was physical activity extracted as covariate?** No

**Results/Conclusion:** “In six of eight Brazilian studies, there was a significant association between watching television and obesity. In one of them, there was also a significant association between watching television and food intake..”

**Salmon J, Tremblay MS, Marshall SJ, Hume C (2011) Health risks, correlates, and interventions to reduce sedentary behavior in young people. Am J Prev Med 41: 197-206.**

**AMSTAR Score - 3**

**Was scientific quality assessed and documented? No**

**Were conclusions formulated based on high methodological quality studies? No**

**Does the conclusions were based in longitudinal studies?** No

**Was physical activity extracted as covariate?** No

**Results/Conclusion:** Children who watch more TV also have higher energy intake from fat, sweet and salty snacks, and high-energy drinks and lower consumption of fruits and vegetables. Positive associations between “unhealthy” food advertising and overweight prevalence and inverse associations between “healthy” food advertising and overweight prevalence have also been reported.”

**Level of Evidence – Insufficient Evidence**

***OTHER SOCIAL BEHAVIOURS – Based on Salmon et al.***

**Salmon J, Tremblay MS, Marshall SJ, Hume C (2011) Health risks, correlates, and interventions to reduce sedentary behavior in young people. Am J Prev Med 41: 197-206.**

**AMSTAR Score - 3**

**Was scientific quality assessed and documented? No**

**Were conclusions formulated based on high methodological quality studies? No**

**Does the conclusions were based in longitudinal studies?** No

**Was physical activity extracted as covariate?** No

**Results/Conclusion:** “There is evidence that high media use clusters with other less-healthy be- haviors. A systematic review examined the effects of all types of media (TV, magazines, music) on tobacco use, drug, and alcohol use, more often sad or unhappy, frequently bored, and had poorer relationships with their parents.

**Level of Evidence – Insufficient Evidence**

***ADULTS***

***SMOKING – Based in Salmon***

**Salmon J, Tremblay MS, Marshall SJ, Hume C (2011) Health risks, correlates, and interventions to reduce sedentary behavior in young people. Am J Prev Med 41: 197-206.**

**AMSTAR Score - 3**

**Was scientific quality assessed and documented? No**

**Were conclusions formulated based on high methodological quality studies? No**

**Does the conclusions were based in longitudinal studies?** No

**Was physical activity extracted as covariate?** No

**Results/Conclusion:** “There is evidence that high media use clusters with other less-healthy be- haviors. A systematic review examined the effects of all types of media (TV, magazines, music) on tobacco use, drug, and alcohol use, more often sad or unhappy, frequently bored, and had poorer relationships with their parents.

**Level of Evidence – Insufficient Evidence**

***ALCOHOL CONSUMPTION AND EATING BEHAVIOUR. – Insufficient Evidence***

**Pearson N, Biddle SJH (2011) Sedentary behavior and dietary intake in children, adolescents, and adults: A systematic review. Am J Prev Med 41: 178-188.**

**AMSTAR Score - 3**

**Was scientific quality assessed and documented? Yes**

**Were conclusions formulated based on high methodological quality studies? Yes**

**Does the conclusions were based in longitudinal studies?**

**Was physical activity extracted as covariate?** No

**Results/Conclusion:** “The association drawn mainly from cross-sectional studies is that sedentary behavior, usually assessed as screen time and predominantly TV viewing, is associated with unhealthy dietary behaviors in children, adolescents and adults.”

**Level of Evidence – Insufficient Evidence**

**OTHER OUTCOMES**

***BONE LOSS – Based on Chinapaw***

**Chinapaw MJ, Proper KI, Brug J, van Mechelen W, Singh AS (2011) Relationship between young peoples' sedentary behaviour and biomedical health indicators: a systematic review of prospective studies. Obes Rev 12: e621-632.**

**AMSTAR Score – 7**

**Was scientific quality assessed and documented? Yes**

**Were conclusions formulated based on high methodological quality studies? Yes**

**Does the conclusions were based in longitudinal studies?** Yes

**Was physical activity extracted as covariate?** Yes - (14/31) = 45%

**Results/Conclusion:** Few studies examined the longitudinal relationship between sedentary time and other health outcomes such as blood pressure, blood lipids, ﬁtness/VO2max or bone mass. Based on these ﬁndings, we can conclude that there is moderate evidence for a longitudinal inverse relationship between sedentary time and aerobic ﬁtness (based on one high-quality and two low- quality studies) and insufﬁcient evidence for a longitudinal relationship between sedentary time and blood pressure, blood lipids or bone mass.

**Level of Evidence – Insufficient Evidence**

***MOTOR DYSFUNCTION – Based on LeBlanc***

**LeBlanc AG, Spence JC, Carson V, Connor Gorber S, Dillman C, et al. (2012) Systematic review of sedentary behaviour and health indicators in the early years (aged 0-4 years). Appl Physiol Nutr Metab 37: 753-772.**

**Was scientific quality assessed and documented? Yes**

**Were conclusions formulated based on high methodological quality studies? Yes**

**Does the conclusions were based in longitudinal studies?** Yes

**Was physical activity extracted as covariate?** No

**Results/Conclusion:** This review has found evidence that increased television viewing is associated with unfavourable measures of adiposity and decreased scores on measures of psychosocial health and motor skill development.

**Level of Evidence – Insufficient Evidence**

***POOR ACADEMIC PERFORMANCE – Based on Tremblay***

**Salmon J, Tremblay MS, Marshall SJ, Hume C (2011) Health risks, correlates, and interventions to reduce sedentary behavior in young people. Am J Prev Med 41: 197-206.**

**Was scientific quality assessed and documented? No**

**Were conclusions formulated based on high methodological quality studies? No**

**Does the conclusions were based in longitudinal studies?** No

**Was physical activity extracted as covariate?** No

**Results/Conclusion:** “Exposure to large amounts of TV can result in poorer cognitive development, short-term memory, academic achievement and language skills, and fewer words in children’s vocabulary. A recent review suggested that flashing lights, quick edits, and auditory cuts from TV may overstimulate developing brains and affect negatively language development, attention span, and cognitive development.

**Level of Evidence – Insufficient Evidence**

**Tremblay MS, LeBlanc AG, Kho ME, Saunders TJ, Larouche R, et al. (2011) Systematic review of sedentary behaviour and health indicators in school-aged children and youth. Int J Behav Nutr Phys Act 8:98. doi: 10.1186/1479-5868-8-98.**

**AMSTAR Score - 10**

**Was scientific quality assessed and documented? Yes**

**Were conclusions formulated based on high methodological quality studies? Yes**

**Does the conclusions were based in longitudinal studies?** Yes

**Was physical activity extracted as covariate?** Yes

**Results/Conclusion:** > 2 hrs of screen time per day is negatively associated with academic achievement. Dose-response relation between time spent playing video games, watching TV and using the computer (for non-academic purposes). > 3 hrs/day associated with poor school performance and lower I.Q. scores.

**Level of Evidence – Strong Evidence**

***PHYSICAL FITNESS – Based on Tremblay***

**Chinapaw MJ, Proper KI, Brug J, van Mechelen W, Singh AS (2011) Relationship between young peoples' sedentary behaviour and biomedical health indicators: a systematic review of prospective studies. Obes Rev 12: e621-632.**

**AMSTAR Score - 7**

**Was scientific quality assessed and documented? Yes**

**Were conclusions formulated based on high methodological quality studies? Yes**

**Does the conclusions were based in longitudinal studies?** Yes

**Was physical activity extracted as covariate?** Yes - (14/31) = 45%

**Results/Conclusion:** Few studies examined the longitudinal relationship between sedentary time and other health outcomes such as blood pressure, blood lipids, ﬁtness/VO2max or bone mass. Based on these ﬁndings, we can conclude that there is moderate evidence for a longitudinal inverse relationship between sedentary time and aerobic ﬁtness (based on one high-quality and two low- quality studies) and insufﬁcient evidence for a longitudinal relationship between sedentary time and blood pressure, blood lipids or bone mass.

**Level of Evidence – Strong Evidence**

**Tremblay MS, LeBlanc AG, Kho ME, Saunders TJ, Larouche R, et al. (2011) Systematic review of sedentary behaviour and health indicators in school-aged children and youth. Int J Behav Nutr Phys Act 8:98. doi: 10.1186/1479-5868-8-98.**

**AMSTAR Score - 9**

**Was scientific quality assessed and documented? Yes**

**Were conclusions formulated based on high methodological quality studies? Yes**

**Does the conclusions were based in longitudinal studies?** Yes

**Was physical activity extracted as covariate?** No

**Results/Conclusion:** Fifteen studies assessed the relationship between time spent engaging in sedentary behaviour and fitness. Increased time spent being sedentary was associated with decreased scores for overall physical fitness, VO2 max, cardiorespiratory fitness, and musculoskeletal fit- ness. An intervention reported that targeting decreased sedentary behaviour lead to increases in aerobic fitness. The level of evidence related to fit- ness was classified as Level 3 with a mean Downs and Black score of 20.9 (standard deviation: ± 2.1), indicating moderate quality of reporting.

**Level of Evidence – Strong Evidence**

***SYMPTOMATIC GALLSTONE DISEASE – Based on Thorp***

**Thorp AA, Owen N, Neuhaus M, Dunstan DW (2011) Sedentary behaviors and subsequent health outcomes in adults a systematic review of longitudinal studies, 1996-2011. Am J Prev Med 41: 207-215.**

**AMSTAR Score - 4**

**Was scientific quality assessed and documented? Yes**

**Were conclusions formulated based on high methodological quality studies?** Yes

**Were the conclusions were based in longitudinal studies?** Yes

**Was physical activity extracted as covariate?** Yes - (34/48) = 71%

**Results/Conclusion**: Sedentary behavior was significantly associated with increased incidence of cardiovascular disease, symptomatic gallstone disease, and mental disorders largely independent of physical activity time. However, caution is warranted in the interpretation of these fındings as the conclusions drawn are based on a limited number of studies. Further studies are required to confırm a longitudinal relationship.

**Level of Evidence – Insufficient Evidence**
