# Supplementary material for: Sedentary Behavior and Health Outcomes: An Overview of Systematic Reviews
Source: PLoS One. 2014 Aug 21;9(8):e105620. doi: 10.1371/journal.pone.0105620 (PMC4140795; doi:10.1371/journal.pone.0105620)
Supplement: File S6 — Aims, main results and use of causative language in systematic reviews of sedentary behavior and health outcomes. (DOCX) [file pone.0105620.s006.docx]

| **SUPPLEMENTARY FILE 5**: Aims, main results and use of causative language in systematic reviews of sedentary behaviour and health outcomes. * | |
| --- | --- |
| **Children and adolescentes** | |
| Costigan et al.[11] | Aim: To investigate the association between recreational screen-based sedentary behavior and the physical, behavioral, and psychosocial health indicators for adolescent girls.  Main results: Screen-based sedentary behavior *is associated* with a range of adverse health consequences, but additional longitudinal studies are needed to better understand the health impacts.  Causative language: Acceptable. |
| Hoare et al.[12] | Aim: To identify and evaluate the empirical literature reporting the relationships between obesogenic risk factors (physical activity, sedentary behaviour, diet and weight status) and depression in adolescents.  Main results: *Relationships* were found between lack of physical exercise, heightened sedentary behaviour, poor diet quality, obese or overweight and depression in adolescence.  Causative language: Acceptable. |
| Rey-López et al.[17] | Aim: To review published studies about sedentary behaviour among children and adolescents and analyse its specific influences on body composition.  Main results: The *causes* of excessive weight gain in children are multi-factorial [13]. With regard to environmental factors, sufficient evidence exists to recommend setting a limit to the time spent watching TV, especially by young children.  Causative language: Qualified casual.  Main results based on randomized controlled trials: No. |
| Salmon et al.[19] | Aim: To examine the evidence of the relationship between sedentary behavior and health risk indicators.  Main results: A growing body of *evidence supports the development of public health recommendations*  to limit the time spent in screen-based behaviors.  Causative language: Qualified causal.  Main results based on randomized controlled trials: No. |
| Chinapaw et al.[20] | Aim: To describe the prospective relationship between childhood sedentary behaviour and health indicators.  Main results: We found insufficient evidence for a longitudinal positive *relationship* between ‘sedentary time’ – mainly TV viewing – and body mass index (BMI) and more specific indicators of fat mass. One high quality and two low quality studies found a significant inverse *relationship* between sedentary time – mainly TV viewing – and aerobic fitness, leading to moderate evidence for this inverse relationship. There was insufficient evidence for a longitudinal *relationship* between sedentary time and blood pressure, blood lipids or bone mass. Our systematic review suggests that there is moderate evidence for a longitudinal inverse *relationship* between screen time and aerobic fitness during childhood.  Causative language: Acceptable. |
| LeBlanc et al.[21] | Aim: To present the best available evidence on the threshold of sedentary behaviour associated with healthy measures of adiposity, bone health, motor skill development, psychosocial health, cognitive development, and cardiometabolic health indicators in infants, toddlers, and preschoolers.  Main results: This review found low- to moderate- quality evidence to suggest that increased television viewing is *associated* with unfavourable measures of adiposity and decreased scores on measures of psychosocial health and cognitive development.  Causative language: Acceptable. |
| Marshall et al.[22] | Aim: To review the empirical evidence of associations between television (TV) viewing, video/computer game use and (a) body fatness, and (b) physical activity.  Main results: A statistically significant *relationship* exists between TV viewing and body fatness among children and youth although it is likely to be too small to be of substantial clinical relevance.  Causative language: Acceptable. |
| Mitrofan et al.[23] | Aim: To collate and determine the quality of research on associations between aggressive content and amount of television viewing or video games playing and aggression in children and young people with behavioural and emotional difficulties.  Main results: This systematic review found insufficient, contradictory and methodologically flawed  evidence on the association between television viewing and video game playing and aggression in  children and young people with behavioural and emotional difficulties.  Causative language: Acceptable. |
| Pearson and Biddle[24] | Aim: To review whether dietary intake is associated with sedentary behavior in young people and adults.  Main results: The association drawn mainly from cross-sectional studies is that sedentary behavior, usually assessed as screen time and predominantly TV viewing, is *associated* with unhealthy dietary behaviors in children, adolescents, and adults.  Causative language: Acceptable. |
| Prentice-Dunn and Prentice-Dunn[25] | Aim: To examine associations of physical activity (PA) and sedentary behavior to childhood overweight and obesity in cross-sectional studies from the last 10 years.  Main results: In general, sedentary behaviors were positively associated with weight status.  Causative language: Acceptable. |
| Rossi et al.[26] | Aim: To identify the influence that watching television has on food intake and obesity in children and adolescents.  Main results: Since time spent watching television is associated with unhealthy food habits and reduced levels of physical activity, it becomes an important factor in the promotion of obesity in children and adolescents.  Causative language: Acceptable. |
| Tremblay et al.[27] | Aim: To determine the relationship between sedentary behaviour and health indicators in school-aged children and youth aged 5-17 years.  Main results: In particular, the evidence suggests that daily TV viewing in excess of 2 hours is associated with reduced physical and psychosocial health, and that lowering sedentary time *leads to* reductions in BMI.  Causative language: Causal.  Main results based on randomized controlled trials: Yes. |
| Te Velde et al.[28] | Aim: To systematically identify dietary, physical activity and sedentary behaviours in preschool children (4–6 years of age) that are prospectively related to overweight or obesity later in childhood.  Main results: Strong evidence was found for an inverse *association* between total physical activity and overweight. Moderate evidence was observed for a positive *association* between television viewing and overweight. Because of the heterogeneity in the assessed dietary behaviours, insufficient evidence was found for an *association* between dietary intake or specific dietary behaviours and overweight.  Causative language: Acceptable. |
| **Adults** |  |
| Teychenne et al.[29] | Aim: To investigate the effect of SB on the risk of depression in adults.  Main results: Evidence for the relationship between SB and risk of depression in adults is limited by methodological weaknesses. However, on balance, this review suggests that SB is associated with an increased risk of depression.  Causative language: Acceptable. |
| Edwardson et al.[30] | Aim: To quantify the association between sedentary behavior and the metabolic syndrome in adults using meta-analysis.  Main results: People who spend higher amounts of time in sedentary behaviours have *greater odds* of having metabolic syndrome.  Causative language: Acceptable. |
| Grontved and Hu[31] | Aim: To perform a meta-analysis of all prospective cohort studies to determine the association between TV viewing and risk of type 2 diabetes, fatal or nonfatal cardiovascular disease, and all-cause mortality.  Main results: Prolonged TV viewing was *associated* with increased risk of type 2 diabetes, cardiovascular disease, and all-cause mortality.  Causative language: Acceptable. |
| Proper et al.[32] | Aim: To systematically review the literature as to the relationship between sedentary behaviors and health outcomes considering the methodologic quality of the studies.  Main results: This review of prospective studies showed moderate evidence for an independent *relationship* between sedentary time and type 2 diabetes. In addition, strong evidence was found for sedentary behavior to be *related* to all-cause and CVD mortality, but not for mortality from cancer.  Causative language: Acceptable. |
| Thorp et al.[34] | Aim: To systematically review and provide an informative synthesis of fındings from longitudinal studies published since 1996 reporting on relationships between self-reported sedentary behavior and device-based measures of sedentary time with health-related outcomes in adults.  Main results: Findings indicate a consistent *relationship* of self-reported sedentary behavior with mortality and with weight gain from childhood to the adult years.  Causative language: Acceptable. |
| Lynch et al.[35] | Aim: To evaluate the research on sedentary behavior and cancer, to summarize possible biological pathways that may underlie these associations, and to propose an agenda for future research.  Main results: Sedentary behavior was *associated* with increased colorectal, endometrial, ovarian, and prostate cancer risk; cancer mortality in women; and weight gain in colorectal cancer survivors.  Causative language: Acceptable. |
| Pearson and Biddle[24] | Aim: To review whether dietary intake is associated with sedentary behavior in young people and adults.  Main results: The association drawn mainly from cross-sectional studies is that sedentary behavior, usually assessed as screen time and predominantly TV viewing, is *associated* with unhealthy dietary behaviors in children, adolescents, and adults.  Causative language: Acceptable. |
| Wilmot et al.[36] | Aim: To examine the association of sedentary time with diabetes, cardiovascular disease and cardiovascular and all-cause mortality.  Main results: Sedentary time is *associated* with an increased risk of diabetes, cardiovascular disease and cardiovascular and all-cause mortality; the strength of the association is most consistent for diabetes.  Causative language: Acceptable. |
| Boyle[37] | Aim: There is also emerging evidence that sedentary behavior, or too much sitting, may be a distinct risk factor for colon cancer that acts independently of physical activity. The aim of this review is to summarize the research that has investigated this issue. |
|  | Main results: Despite these methodological problems, the research conducted in this area so far indicates that sedentary behavior *may be* a risk factor for colon cancer, independently of physical activity. |
|  | Causative language: Qualified causal. |
| Ford and Caspersen[38] | Aim: To examine the relationship between sedentary behaviour and cardiovascular morbidity and mortality using prospective observational studies conducted largely during the past decade.  Main results: The majority of prospective studies of screen time and sitting time has shown that greater sedentary time is *associated* with an increased risk of fatal and non-fatal CVD.  Causative language: Acceptable. |
| Chen et al[39] | Aim: To review systematically studies examining the association between sedentary lifestyle and low back pain (LBP) using a comprehensive definition of sedentary behaviour including prolonged sitting both at work and during leisure time.  Main results: The present review confirms that sedentary lifestyle by itself is not associated with LBP.  Causative language: Acceptable. |
| IJmker et al[41] | Aim: This systematic review summarises the evidence for a relationship between the duration of work time spent using the computer and the incidence of hand–arm and neck–shoulder symptoms and disorders.  Main results: Moderate evidence was concluded for a positive *association* between the duration of mouse use and hand–arm symptoms. For this association, indications for a dose–response relationship were found.  Causative language: Acceptable. |
| van Uffelen et al[42] | Aim: To systematically review the evidence on associations between occupational sitting and health risks.  Main results: Limited evidence was found to support a positive *relationship* between occupational sitting and health risks.  Causative language: Acceptable. |
| Waersted et al[43] | Aim: This review examines the evidence for an association between computer work and neck and upper extremity disorders (except carpal tunnel syndrome).  Main results: There is limited epidemiological evidence for an association between aspects of computer work and some of the clinical diagnoses studied.  Causative language: Acceptable. |
| Teychenne et al.[44] | Aim: The current literature review aims to systematically examine the evidence investigating the association between physical activity, sedentary behavior, and postnatal depressive symptoms, focusing particularly on the dose and domain in which these behaviors are undertaken. |
|  | Main results: There is limited evidence suggesting that there are positive *associations* between sedentary behavior and postnatal depressive symptoms. |

*For reviews that used a causal or qualified causal language in their results, we show whether they included randomized controlled trials were or not.
